# Changes and drivers of bacterioplankton communities within plain river networks during the rainy season (high inflow event): simulation of the water level using the MIKE11 model

**DOI:** 10.1128/aem.01124-25

**Published:** 2025-11-28

**Authors:** Jun Zhao, Thomas Hein, Lachun Wang

**Affiliations:** 1 School of Geography and Ocean Science, Nanjing University 12581, Nanjing, China; 2 Institute of Hydrobiology and Aquatic Ecosystem Management, BOKU University 27270, Vienna, Austria; 3 School of Civil Engineering and Transportation, South China University of Technology 26467, Guangzhou, China; 4 Christian Doppler Laboratory for Meta Ecosystem Dynamics in Riverine Landscapes, BOKU University 27270, Vienna, Austria; Colorado School of Mines, Golden, Colorado, USA

**Keywords:** rainy season, bacterioplankton, river, network analysis, hydrodynamic model

## Abstract

Plain river networks are becoming increasingly sensitive and vulnerable under the combined effects of climate change and landscape alteration. Understanding adaptive changes in bacterioplankton communities in response to the environment is important for improving the health of river networks. Still, little is known about the changes in bacterioplankton communities and their drivers during the rainy season. By applying co-occurrence networks, source-sink analyses, variance partitioning, and the MIKE11 model, we investigated changes in the composition, interaction, and source of the bacterioplankton community at 32 sampling sites along an anthropogenic pressure gradient (urban, suburban, and agricultural area) in the Taihu Lake plain river networks before and after the Plum rain (East Asian rainy season), as well as the drivers (water chemistry, land use, and hydrological factors) of this change. The results showed that the Chao1 diversity index and Shannon diversity index (richness) decreased significantly, and the inverse Simpson’s diversity index (evenness) increased significantly after the plum rain, especially in the suburban area, indicating a decrease in the contribution of rare taxa. Related to the reduction of inputs by rare taxa after the plum rain, network analyses revealed that bacterioplankton communities were no longer tightly linked. The analysis demonstrated that water chemistry explained these changes in the cyanobacterial communities better than the hydrological indicators as evidenced by the substantially higher explanatory power of simulated data (pre-plum rain: 86%; post-plum rain: 50%) compared to measured data (pre-plum rain: 36%; post-plum rain: 22%). The use of models to obtain hydrological data was feasible when the integrity of the data analysis was ensured and the analytical process was optimized.

**IMPORTANCE:**

Unlike natural rivers, the microbial response mechanisms in human-dominated plain river networks remain poorly understood. These densely populated aquatic ecosystems, under intense anthropogenic influence, face dual pressures from climate change and landscape alteration. Their fragile hydrodynamics increase ecological vulnerability during the rainy season. Focusing on the Plum rain season, this study reveals that after the rainfall, community diversity significantly decreases, the contribution of rare taxa reduces, and bacterioplankton communities were no longer tightly linked. Combining MIKE11 modeling with multi-source environmental data, the study clarified the decisive role of water chemistry parameters in Cyanobacteria community shifts and validated the applicability of model data in ecological process research. These findings advance understanding of microbial adaptation in human-disturbed river networks and support ecosystem health assessment. The model-data fusion analysis method established in this study provides a technical framework for similar water ecosystem research.

## INTRODUCTION

Climate change and landscape alteration are occurring on an unprecedented scale globally, exerting enormous pressure on river ecosystems (1, 2). Particularly in the plain river network area, which is densely populated and intensively used, it suffers from the pressure of intense anthropogenic disturbances (3, 4); at the same time, the area is weak in hydrodynamic conditions and prone to flooding during the rainy season and suffers from the risk of meteorological disasters (5–7). This has resulted in the area showing growing issues of species loss, pollution from internal and external sources, eutrophication, and others (8–11). The sensitivity of bacterioplankton communities to changes in environmental conditions makes them one of the pioneering aquatic organisms responding to the physico-chemical environment of river ecosystems (12, 13), i.e., makes them key indicators of changes in the conditions described above (4, 14). Therefore, investigating adaptive changes in bacterioplankton communities in plains river networks is critical to understanding how ecosystems maintain their functions and services in the face of climate change (15).

Based on various anthropogenic disturbances, such as land development (4, 14), industrialization (16, 17), hydraulic engineering (18, 19), bacterioplankton communities tend to form different ecological communities or modular organizations, which interact to maintain community stability (20, 21), demonstrating the influence of land use change, water chemistry, and hydrology on community network structure. In addition, it has been found that community network structure varies with seasonal rainfall changes (22, 23): rainfall facilitates the connection of runoff from the riparian land to the water bodies of the river network, inducing changes in the network biologically (introduction or elimination of species), chemically (addition of nutrients or pollutants), and physically (diffusion or convection). Recent work has further elaborated on these mechanisms, showing how rainfall events can rapidly reconfigure microbial networks (24–26). Such past studies have provided insights into the effects of long-term climatic disturbances on riverine network communities of microorganisms; however, it is unclear how the structure of bacterioplankton networks under the pressure of anthropogenic disturbances will respond to changes in the rainfall environment. Notably, plain river networks are subject to runoff and tidal influences, which complicates understanding the network structure of bacterioplankton communities during the rainy season.

Currently, numerical modeling techniques have been widely applied to flood management and the prediction of plain river networks (27–29). Its continuous development has enabled us to get rid of spatial and temporal constraints, extend monitoring boundaries, save monitoring costs, and achieve an understanding of the overall general pattern of plain river networks, where the MIKE11 hydrodynamic modeling has become an outstanding representative of urban flood control science in the European plains. Previous studies have described the hydrodynamic conditions of water bodies through hydrodynamic models to quantify plain river networks with high spatial and temporal resolution during the rainy season (high flow events) (6, 30, 31) but have not further elaborated the physiological and ecological patterns of aquatic organisms, e.g., fish (32), phytoplankton (33), and microorganisms (34). Wang et al. (32) applied MIKE to simulate changes in water temperature to assess areas of fish spawning; Schuchert et al. (33) modeled the effects of changes in coastal hydrodynamics on phytoplankton by MIKE; and Li et al. (34) applied MIKE to quantify the contribution of the river fluid environment to the nitrogen concentration of microbial participation. However, these studies have not considered bacterioplankton communities responding to the hydrodynamic conditions of plain river networks at large spatial scales. Furthermore, given the scarce data sets and spatial and temporal coverage of existing studies, it is impossible to identify a common thematic pattern controlling the distribution of bacterioplankton communities in rivers. This knowledge is becoming increasingly important as many rivers are under threat from increasing high-flow events with global climate change (35–37), and our information on the patterns of interactions between bacterioplankton accompanied by high freshwater inflow events, such as intense rainfalls, is still limited.

In the lower reaches of the Yangtze River, Asia’s longest river, a period of extremely wet weather, occurs due to the control of the western Pacific subtropical high. Under this weather system, eastern China experiences persistent rainfall that can account for 40%–50% of total precipitation during the rainy season, known as plum rain. This climatic feature provides a unique context for our study of changes in bacterioplankton communities during the rainy season. At this time, eastern China is suffering from severe blooms, and as a result, many experts have carried out extensive work on algae (9, 11). However, investigations of bacterioplankton communities, as the basis of riverine food webs, have been sparsely documented in the context of rainy season in plain river networks.

Given the current state of research, we investigated plain river networks in urban, suburban, and agricultural areas along an anthropogenic pressure gradient during the rainy season, aiming to understand the ecological network of bacterioplankton communities in the river networks during this period; identify species that differ significantly before and after the rainy season; and elucidate the factors influencing changes in bacterioplankton communities following the spatial parameterization of hydrodynamic conditions in a plain river network using the MIKE11 model (Fig. S1).

## MATERIALS AND METHODS

### Study area

**Fig 1:**
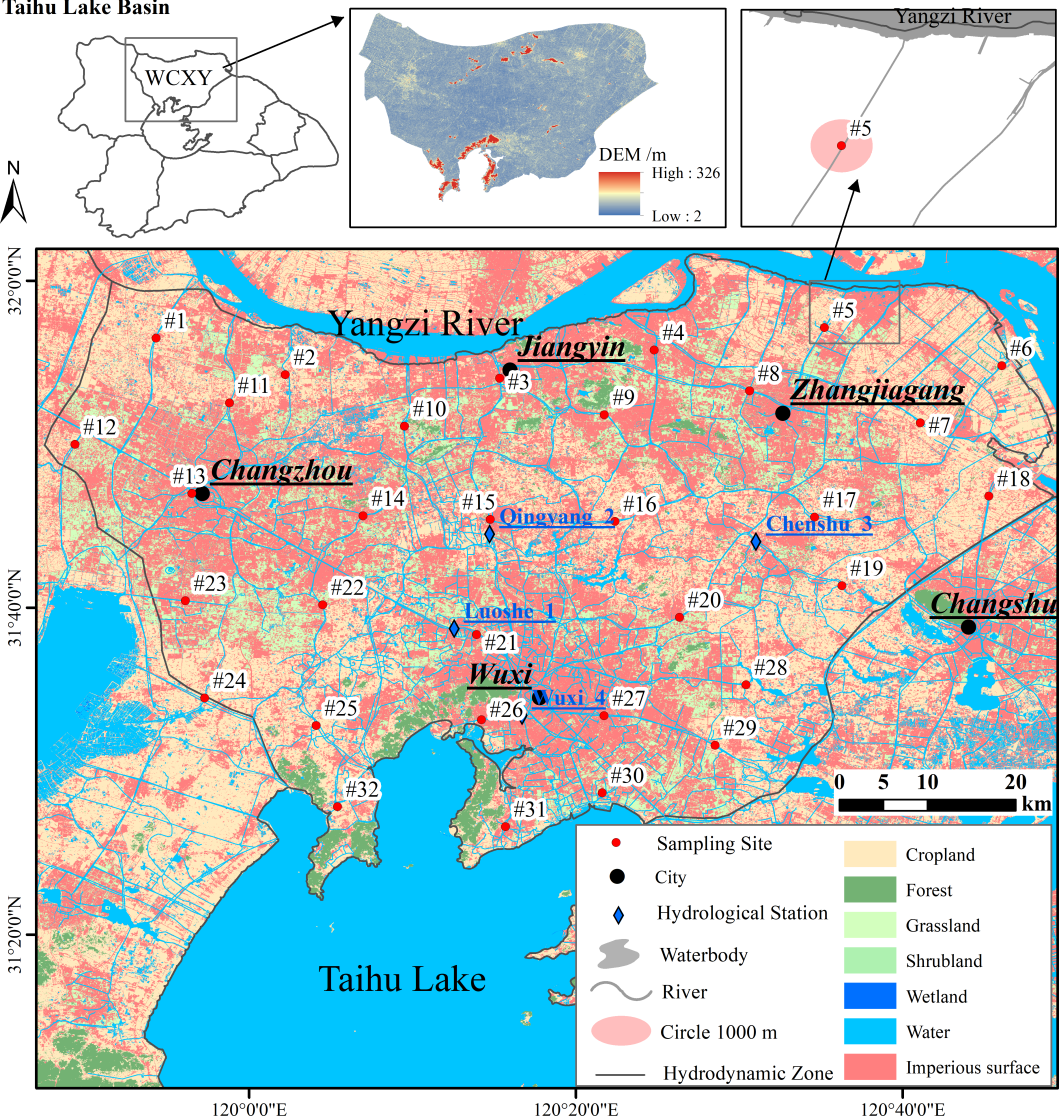
Sampling sites and their circular buffer zones within the WCXY hydrodynamic zone. The base map was sourced from OpenStreetMap, land use data were derived from 10 m resolution Sentinel-2 satellite imagery [https://data.ess.tsinghua.edu.cn/], and the underlying Digital Elevation Model (DEM) data were obtained from the 30 m resolution Copernicus DEM.

Wuchengxiyu hydrodynamic zone (WCXY) is located in a low-lying plain in the north of the Taihu Lake basin (Fig. 1). WCXY borders the Yangtze River to the north and Taihu Lake to the south, making it play a crucial role as a significant hydrodynamic zone ensuring water quality for the Yangtze River and Taihu Lake.

WCXY belongs to the subtropical monsoon zone. The flood season is from June to September, and the precipitation is bimodal (long rainy season from June to August and a short rainy season caused by typhoons in September). During the relatively stable stage of the East Asian summer monsoon, the rainfall is evenly distributed with continuous heavy rainfall, which can last for 2 months. At this time, the water level in the low-lying river network area is high, and the risk of flooding disasters is large, which is generally called the “plum rain” (also known as Meiyu in China, Tsuyu in Japan, and Jangma in Korea). From mid-June to mid-August 2022, a total of 6 heavy rain (25–50 mm per 24 h) events and one rainstorm (>50 mm per 24 ho) event was recorded according to the Chinese precipitation classification standard (GB/T 28592-2012). The total rainfall ranged from 184.80 to 331.70 mm (from two meteorological stations in WCXY: Wuxi and Zhangjiagang) accounting for 20.69% to 33.60% of the annual rainfall (Fig. S2).

Since the combination of natural discharge, tidal effects, and anthropogenic management results in the bi-directional flow of rivers in this area, the land use pattern of the circular buffer zone around each sampling site was considered to be the most important riparian environment in the study area (38). The scale of the buffer zone was determined according to a previous study (4), which found that the 1,000 m buffer zone was the most effective scale influencing the bacterioplankton community in this area, based on a statistical comparison of the explanatory power (Mantel test) of land use patterns across multiple buffer scale (e.g., 100 m, 300 m, 500 m, and 1,000 m). For the effective analysis, ArcToolbox (ArcGIS v.10.2) was used to calculate the proportion of land use patterns within the circular buffer zone around the 32 sampling sites and classified them into the three types of area (Fig. S3), namely, urban area (*n* = 8, No. #3, #4, #13, #14, #15, #21, #27, #31, impervious surface exceeded 70%); agricultural areas (*n* = 14, No. #1, #2, #5, #6, #7, #16, #17, #18, #19, #24, #25, #28, #29, #32, cropland exceeded 50%); and others were suburban areas (*n* = 10, No. #8, #9, #10, #11, #12, #20, #22, #23, #26, #30, mixed forests and wetland parks).

### Observational data and measurement of physicochemical parameters

A total of 32 sampling sites were evenly distributed across WCXY river networks. Sampling had 4–7 days of no rain fall prior to avoid immediate disturbances from long-term rainfall. A sterilized water sample collector (Suzhou Air Instrument Co., China) was used to collect surface water samples at each sampling point on the bridge (approximately 0.5 m below the surface water) in triplicate. Two abbreviations Jun.A and Aug.B represent the two sampling rounds in June and August, respectively, implying pre- and post- plum rain. Officially, the plum rain in the Taihu Lake basin was designated from 12 June to 1 July 2022 [Source: Taihu Basin Authority of Ministry of water resources, ].

Water temperature (*T*), dissolved oxygen (DO), pH, and electrical conductivity (EC) were measured *in situ* with the Y86031 probe (YHequipment Co, China). At each sampling site, three 1 L water samples were collected and pooled to form a single composite sample. This composite sample was then filtered for subsequent DNA extraction and sequencing to obtain a representative profile of the bacterioplankton community. All samples were stored in low-temperature incubators (0–4°C) and transported to a nearby laboratory for sample processing within 4 h after collection. Water depth and flow rate were measured on-site using a tape measure and a current meter (LS1206B, China).

Water samples from each sampling site were divided into five 50 mL sterile centrifuge tubes. Among them, (a) dissolved organic carbon (DOC) was measured by total organic carbon automatic analyzer (Shimadzu TOC-L CPH, Japan); (b) Anions, chloride (Cl^−^), sulfate (SO_4_^2−^), and fluoride (F^−^) were measured by ion chromatography (Dionex ICS2100, United States); (c) Cations, potassium (K^+^), calcium (Ca^2+^), sodium (Na^+^), and magnesium (Mg^2+^) were measured by ICP-OES (PerkinElmer, United States); (d) analysis of ammonia nitrogen (NH_4_-N), nitrite nitrogen and nitrate nitrogen (NOx-N), phosphate phosphorus (PO_4_-P) and (e) analysis of total nitrogen (TN) and total phosphorus (TP) were measured using a continuous flow automatic analyzer (San++, Skalar, Netherlands). Except for TN and TP analyses, all water samples were filtered through a glass fiber filter membrane with a pore size of 0.45 µm (diameter 25 mm, China).

### Molecular analysis

Obtained sample (1 L) was filtered through a 0.22 μm-aperture filter membrane (50 mm diameter, China) to collect microbial cells, and the sample was passed through multiple filters at the same time to reduce filtration time. The collected samples are stored in a sterile centrifuge tube at −80 ° C until DNA extraction. Detailed experimental procedures can be found in the supplementary file. The raw data were generated by Illumina’s MiSeq platform PE3000 and uploaded to NCBI Sequence Read Archive (accession number: SRP448176).

The DADA2 method in the Qiime2 (v.2022.2) process was used to reduce the noise of the optimized sequences after quality control concatenation (39). The sequence obtained from DADA2 noise reduction was called Amplicon Sequence Variant (ASV), and the ASV representative sequence and abundance table were obtained.

Based on the Sliva 16S rRNA database (v.138), the Naivebayes classifier in the Qiime2 process was used to conduct taxonomic analysis of ASVs—domain, kingdom, phylum, class, order, family, genus, and species. The classification confidence was 0.7. TaxAss was used to assign ASVs to the FreshTrain freshwater bacteria database for additional classification and allocation of bacterioplankton communities (40, 41). ASVs inconsistent with the FreshTrain database were classified as “unclassified.”

Since the impact of rare taxa on the within sample (alpha) diversity estimate cannot be ignored in plain river network area (4), we calculated the Hill numbers (iNEXT R Package v.0.5.1) based on the abundance and relative abundance weights of different species (42). Detailed formula calculations can be found in the supplementary file. ^0^D indicates observed species (Sobs); ^1^D indicates the Shannon diversity; ^2^D represents the Inverse Simpson’s index, emphasizing the abundant taxa and downplaying the contribution of rare taxa to diversity estimate. The higher value means the higher species evenness distribution.

### Statistical analysis of community drivers and assembly processes

To establish an understanding of the forces structuring the bacterioplankton community, our analysis moved from identifying broad environmental drivers to resolving fine-scale ecological interactions and temporal dynamics.

The relative importance of water chemical variables and land use on α-diversity was determined using the randomForest algorithm (R package v.4.7-1.1) based on 1,000 decision trees (43). To ensure a robust and reliable assessment of the model’s predictive performance, a 5-times repeated 10-fold cross-validation was employed. This process involves randomly partitioning the data into 10-fold five separate times, each time performing a full 10-fold cross-validation cycle. The final reported accuracy represents the mean accuracy across all 5 repeats and 10 folds (i.e., 50 model fits in total), which provides a more stable and generalizable performance estimate. Regression of variables and diversity values was performed, and each variable was ranked by importance according to its relative contribution to the prediction accuracy of the model.

Beyond the influence of these environmental measured variables, the microbial co-occurrence network was investigated to infer potential ecological interactions and identify keystone taxa (21). The topological properties of a network include degree, betweenness centrality, closeness centrality, eigenvector centrality, clustering coefficient, path length, density, and modularity (44). Calculate each topology parameter in the web page (http://ieg4.rccc.ou.edu/MENA/). The Gephi (v.0.9.2) was used to visualize the co-occurrence network. Modules—groups of highly interconnected nodes reflecting habitat niches—were identified. Each node was assigned a topological role based on its within-module connectivity (*Z*_*i*_) and among-module connectivity (*P*_*i*_) values: peripherals (*Z*_*i*_ ≤ 2.5, *P*_*i*_ < 0.62), module hubs (*Z*_*i*_ > 2.5, *P*_*i*_ ≤ 0.62), connectors (*Z*_*i*_ ≤ 2.5, *P*_*i*_ > 0.62), and network hubs (*Z*_*i*_ > 2.5, *P*_*i*_ > 0.62). The latter three roles are collectively considered keystone taxa.

To directly quantify the temporal legacy of the initial community and the influx from external sources, we employed the FEAST source tracking framework (R package v.0.1.0) (45). This analysis assessed the proportion of the bacterioplankton community in August (Aug.B) that originated from the June community (Jun.A), which was treated as a source. The remaining, unattributed proportion signifies the combined influence of unknown sources, such as exogenous inputs or immigration from unsampled environments.

The analyses above identified key environmental correlates and temporal dynamics of the bacterioplankton community. However, to move beyond statistical correlation and toward a mechanistic understanding of how the hydrological regime shapes these ecological patterns, we employed a one-dimensional hydrodynamic model.

### Hydrological modeling and integration with ecological data

To simulate the hydrological conditions that directly influence the dispersal and mixing of bacterioplankton, we constructed a one-dimensional hydrodynamic model using MIKE11 HD. The plain river network provides an excellent system for such modeling due to its free-surface flow and well-documented artificial channels. The model domain was built using data from the Taihu Lake Basin water system planning map and Google Earth as reference, resulting in a network comprising 131 river channels, 54 sluices, and 46 water level boundaries (Fig. S4).

The MIKE11 HD model was calibrated and validated using observed water level data from four national hydrological stations (Luoshe_1, Qingyang_2, Chenshu_3, and Wuxi_4; Fig. 1). The primary parameter adjusted during manual calibration was the Manning roughness coefficient (*n*). The initial value range (0.025–0.032) was selected based on empirical parameters established for Taihu Lake basin rivers in prior research. During the dry-season calibration phase (7 February to 11 March 2022, with 0 mm of recorded rainfall), iterative parameter adjustments were performed to minimize the deviation between simulated and observed water levels, ensuring the model remained unaffected by rainfall-runoff processes (Fig. S5). The simulation period for this study spans from 09:00:00 on 13 June 2022 to 09:00:00 on 26 August 2022, with a daily time step (Fig. S6). Detailed formula calculations for the MIKE11 HD model can be found in the Supplemental material.

Model accuracy was quantitatively assessed using the Nash-Sutcliffe efficiency coefficient (NSE). Simulation results for all monitoring stations were classified as either good (NSE ≥ 0.75) or satisfactory (0.36 < NSE < 0.75). Detailed comparisons of simulated water levels vs observed water levels at each station are presented in Fig. S5 and S6.

The “bioenv” function was used to obtain the combination of environmental factors with the highest correlation coefficient with the bacterioplankton community (46). The Mantel test was used to determine the correlation and significance level between the bacterioplankton community and the water chemistry and hydrological indicators. This analysis was calculated by vegan (R package v.2.5-7).

The effects of environmental factors on bacterioplankton communities were quantified using VPA analysis. Since VPA analysis does not require non-collinear input of environmental factors (46, 47), the combination of environmental factors with the highest correlation coefficient was obtained by using the “bioenv” function and input into the VPA quantitative model to obtain a larger explanatory quantity. This analysis was performed using the “varpart” function in the rdacca.hp (R package v.1.0-8) to partition community variation.

## RESULTS

### Taxonomic composition and interactions between taxa

Based on high-throughput sequencing, a total of 2,838,493 high-quality sequences were obtained from the 64 samples, with an average sequence length of 415 bp. The rarefaction curves showed that the current sequencing depth is sufficiently representing the bacterioplankton community in the plain river network area based on the saturation of observed species (Sobs) (Fig. S7).

The bacterioplankton communities were dominated by the Proteobacteria, Actinobacteria, Bacteroidota, and Cyanobacteria, which accounted for more than 90% of the abundance of all species (Fig. S7A). In Jun.A, Proteobacteria (39.41%, only α and γ Proteobacteria) was the most abundance pylum, followed by Actinobacteria (30.62%), Bacteroidota (11.56%), Cyanobacteria (9.99%), and Acidinobacteria (1.63%). In Aug.B, a shift occurred: the abundance of Actinobacteria became the highest (35.19%), followed by Proteobacteria (30.62%), Cyanobacteria (21.45%), Bacteroidota (10.5%), and Acidinobacteria (1.06%). Notably, the relative abundances of Proteobacteria and Bacteroidota decreased significantly in Aug.B, while Actinobacteria and Acidobacteria showed no significant change.

**Fig 2:**
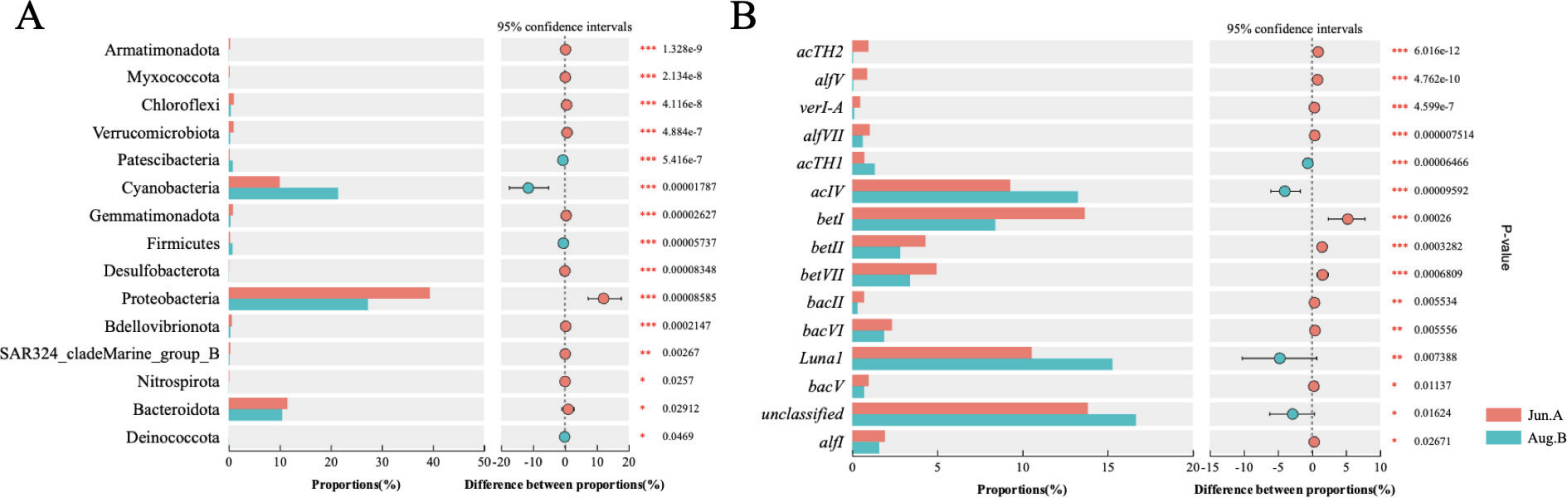
Differential distribution of (**A**) bacterioplankton and (**B**) freshwater bacterioplankton in Jun.A and Aug.B. Red bars and green bars represent the proportion of abundance in Jun.A (pre-plum rain) and Aug.B (post-plum rain), respectively.

This compositional shift was particularly driven by a substantial increase in cyanobacterial abundance after the rain event. Cyanobacteria abundance ranged from 0.88% to 62.09% in Jun.A but increased significantly to a range of 2.16% to 52.28% in Aug.B (Fig. S8C). The phylum was primarily dominated by the class Cyanobacteriia (increasing from 9.95% to 21.36%), with minor contributions from Sericytochromatia and Vampirivibrionia. Beyond the dominant phyla, other taxa such as Armatimonadota, Myxococcota, Chloroflexi, and Verrucomicrobiota exhibited significant decreases in abundance, while Patescibacteria increased significantly in Aug.B (Fig. 2A).

Freshwater bacteria were the dominant lifeform in the WCXY river networks (84.74%, Fig. S8C), and their composition also changed significantly during the rainy season (Fig. 2B). Overall freshwater bacterial abundance decreased in Aug.B, primarily due to reductions in betI (A: 13.67% ± 5.96%; B: 8.41% ± 5.17%) and betVll (A: 4.96% ± 2%; B: 3.40% ± 1.30%) from γ Proteobacteria. The Verrucomicrobiota branch nearly disappeared. However, not all freshwater groups declined; the highly abundant acI (Actinobacteria) remained stable, and the Luna1 (Actinobacteria branch, A: 10.56% ± 11.7%; B: 15.29% ± 10.06%) and acIV (Actinobacteria branch, A: 9.27% ± 4.22%; B: 13.26% ± 4.57%) significantly increased in Aug.B (*P* < 0.01).

**Fig 3:**
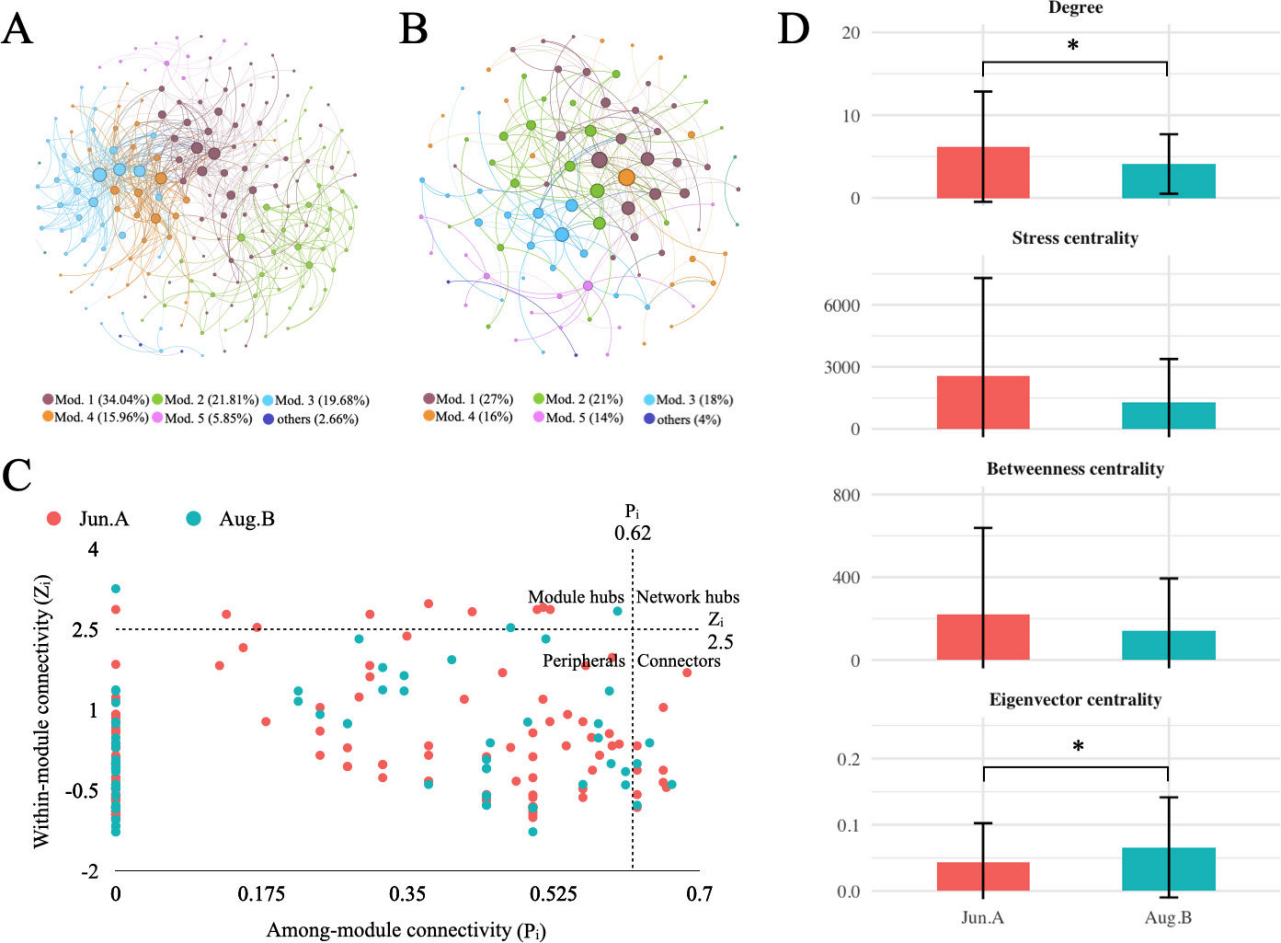
Molecular ecological networks of bacterioplankton communities in Jun.A (pre-plum rain) and Aug.B (post-plum rain). Co-occurrence networks (**A**) in Jun.A and (**B**) in Aug.B. Modules were indicated using different colors. (**C**) Topological roles of bacterioplankton. Each dot represents an ASV. Red and green dots refer to samples from Jun.A and Aug.B, respectively. (**D**) Topological properties for bacterioplankton communities in Jun.A and in Aug.B.

Concurrent with these taxonomic shifts, the co-occurrence networks of the bacterioplankton community underwent simplification after the plum rain (Fig. 3 and Table 1). The network in Jun.A was more complex, with 159 edges connecting 85 nodes, and exhibited the characteristics of a small-world network (SW > 1). In contrast, the Aug.B network was sparser (32 edges connecting 39 nodes) and lost its small-world properties. This was reflected in lower values for degree, stress centrality, and betweenness centrality, but a higher eigenvector centrality in Aug.B, indicating that the network structure became more centralized around a few key nodes. Furthermore, topological role analysis revealed that no keystone species (network hubs) were identified in either period. The vast majority of nodes (>92%) in both networks were peripherals, with the remaining nodes acting as connectors (Fig. 3C and Table S1).

**TABLE 1:** Topological properties of the bacterioplankton molecular ecological networks^*a*^^,^^*b*^

Network properties	Jun. A		Aug. B	
	Empirical networks	Random networks	Empirical networks	Random networks
Total nodes	188	N/A	100	N/A
Total edges	582	N/A	205	N/A
Average degree	6.19	N/A	4.10	N/A
Graph density	0.03	N/A	0.04	N/A
Modularity	0.51	0.33	0.52	0.43
Average clustering coefficient (C)	0.13	0.10	0.04	0.06
Average path length (L)	3.50	3.00	4.09	3.24
Small-world coefficient (SW)	1.14	N/A	0.51	N/A

^*a*^ SW = (C/Cr)/(L/Lr), r is random networks.

^*b*^ “N/A” indicates not available.

### Taxonomic diversity and driving environmental factors

**Fig 4:**
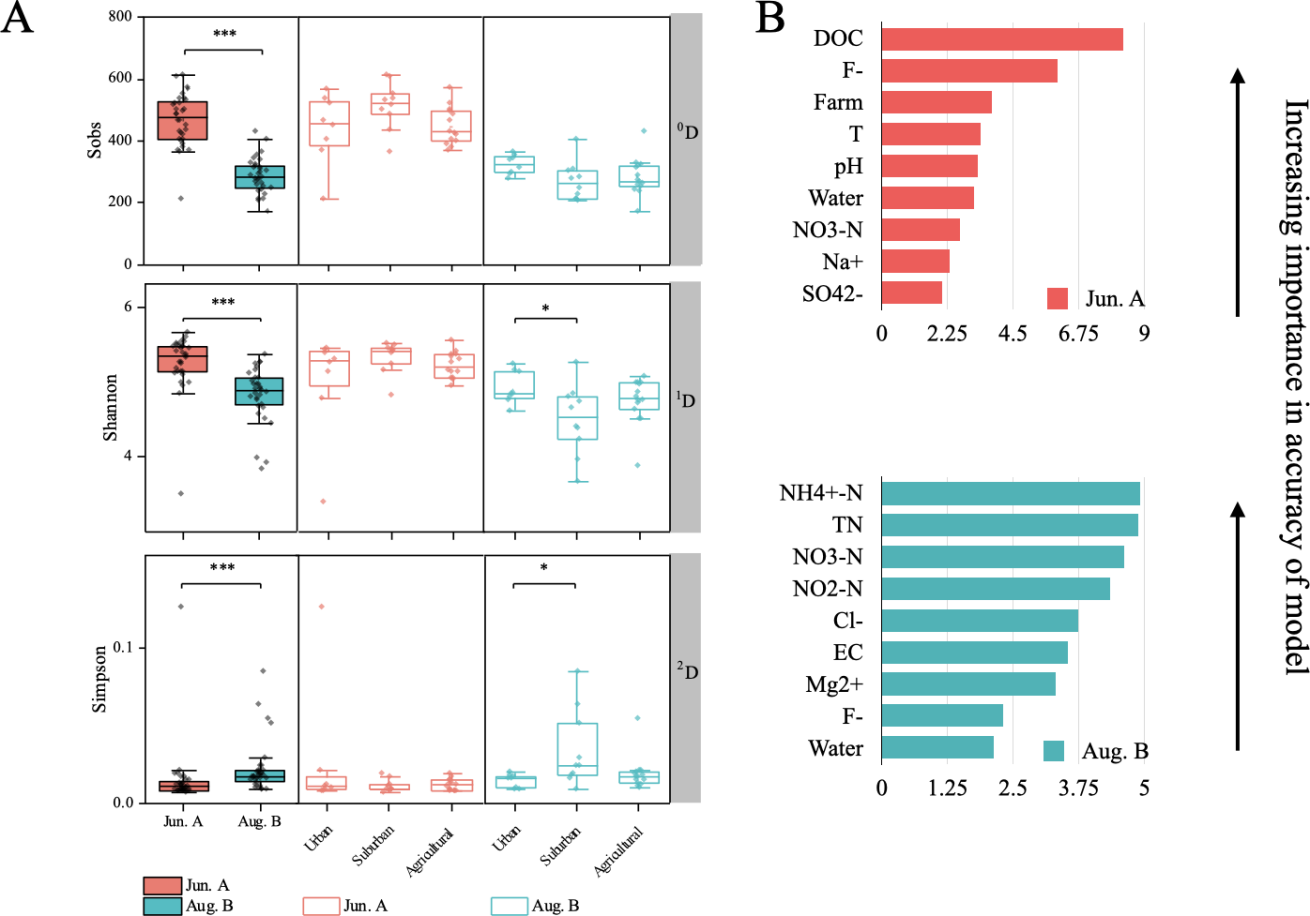
Diversity of bacterioplankton communities and the proportion of variables contributing to observed species. (**A**) Alpha diversity index of the bacterioplankton communities across the land use types in Jun.A (pre-plum rain) and in Aug.B (post-plum rain). (**B**) Random Forest model to evaluate the importance of water chemistry and land use on the Sobs. ^0^D = Observed species; ^1^D = Shannon diversity; ^2^D = Simpson’s index. ***P < 0.001, **P < 0.01, *P < 0.05.

A random forest model, evaluated through a robust 5-time repeated 10-fold cross-validation approach, was employed to assess the importance of land use and water chemistry on the bacterioplankton community α-diversity (represented by species richness, Sobs) in the river network during the rainy season (Fig. 4). The model performance exhibited variability across validation runs (mean accuracy = 0.69 ± 0.3), indicating that the predictability of species richness based on these environmental variables was not stable. It was found that when land use and water chemistry were used as explanatory variables to predict species richness (Sobs). The degree of explainability was 23.81% before the rainy period and 11.67% after the rainy period, and the ability to predict species richness (Sobs) decreased. Before the rainy period, *T* can be a top five environmental factor. After the rainy period, *T* could not be ranked (<1%) in terms of its ability to explain, and if the diversity of rich species is emphasized, the effect of temperature is weak compared to nitrogen nutrients.

**Fig 5:**
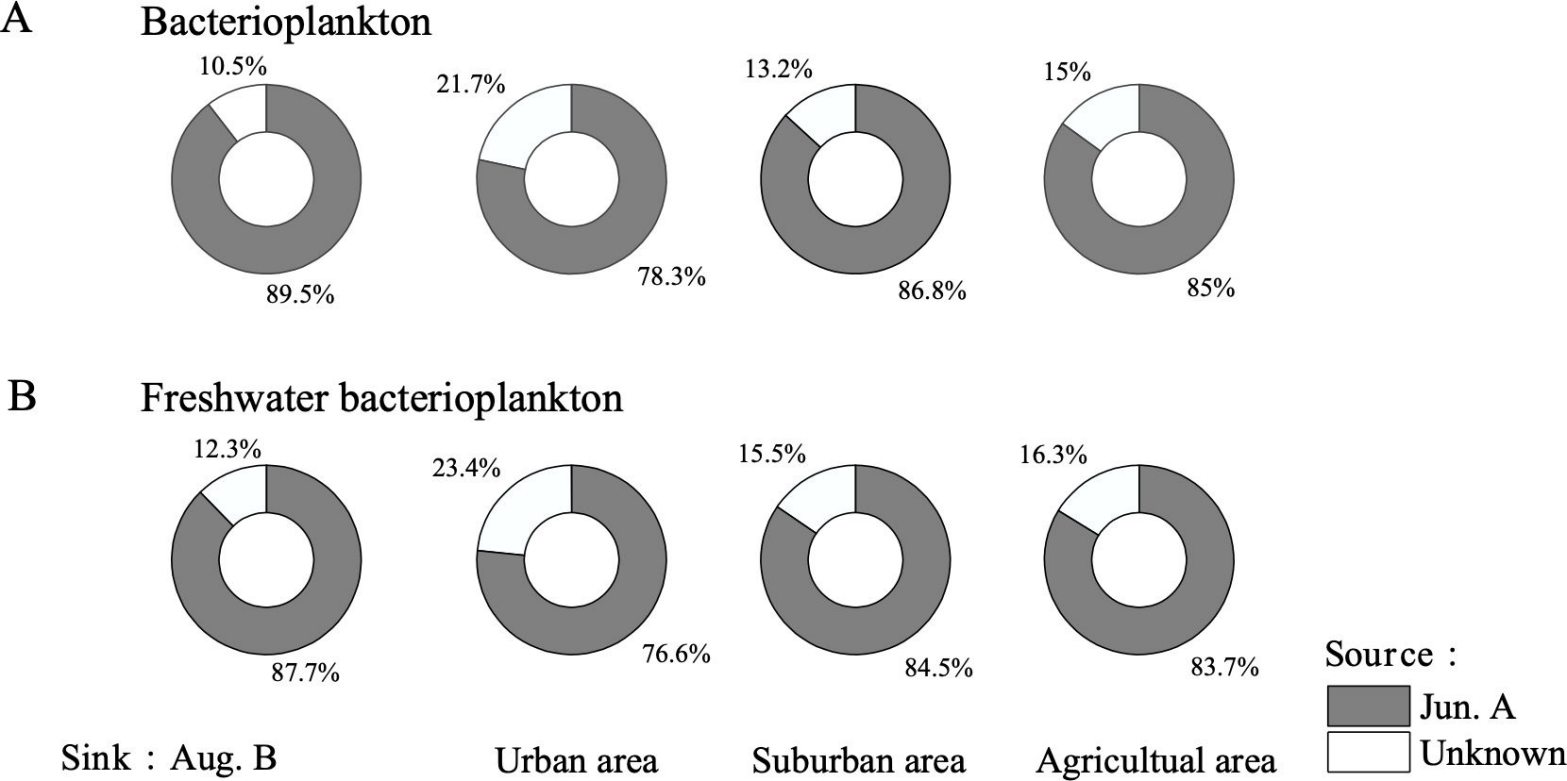
Proportion of source environment (Jun.A) assigned to sink samples (Aug.B). In Aug., (**A**) bacterioplankton and (**B**) freshwater bacterioplankton were from Jun. A, which varied across the land use.

The provenance of bacterioplankton communities in different land-use types after the plum rain revealed that over 87% of the community was derived from the pre-plum rain (Jun.A) period, with a relatively small contribution from unknown sources (Fig. 5).

### Results of the integration of water chemistry and hydrological impacts

**Fig 6:**
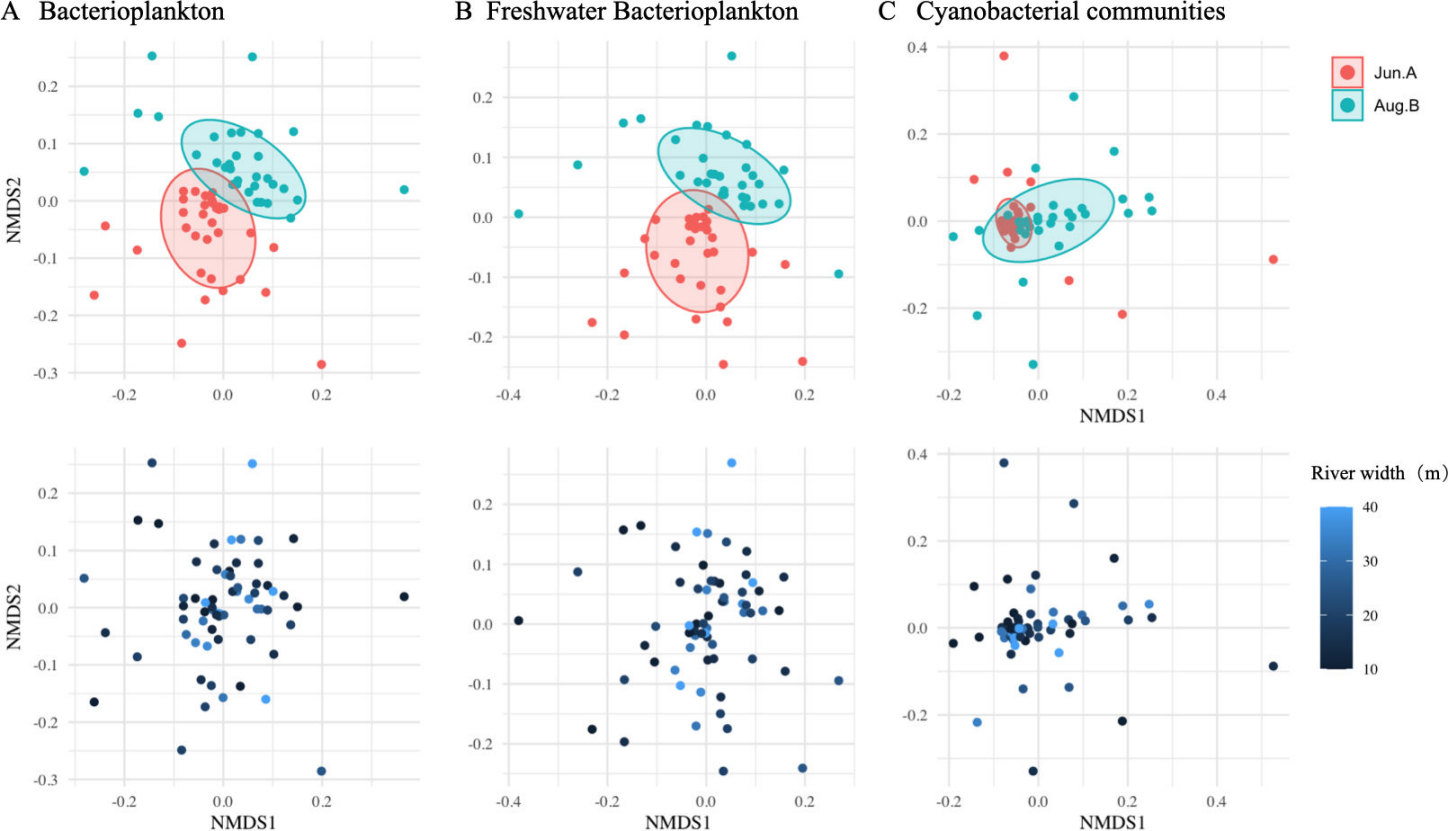
Non-metric multidimensional scaling plots (NMDS) based on Bray-Curtis distances. NMDS plots derived showing (**A**) bacterioplankton, (**B**) freshwater bacterioplankton, and (**C**) cyanobacterial communities before (Jun. A) and after (Aug. B) the Plum rain season and along the river width gradient. Top and down are the same NMDS plots: top panel, plots are color-coded by sampling month in Jun.A (red) and in Aug.B (green) and labeled by site; down panel, river width is depicted with shades of blue, with wider rivers appearing lighter and thinner river appearing darker.

The overall bacterioplankton community structure separated into two distinct groups corresponding to the pre- and post-plum rain periods (Fig. 6A). Cyanobacterial communities from wider rivers formed a distinct cluster prior to the rain (Jun.A), a pattern that disappeared afterward (Fig. 6C). In contrast, no clear patterning was found in the composition of freshwater bacterioplankton along the environmental gradient.

Environmental factors include water chemistry (Fig. S9), land use (Fig. 1B), and hydrological indicators. In Aug.B, the correlation of cropland to nitrogen and phosphorus nutrients decreased, and the correlation to anions and cations shifted from a positive correlation in Jun.A to a negative correlation in Aug.B (Table S2). Forest showed a negative correlation with all water chemistry parameters. The correlation with cations (EC) increased in Aug.B, while the correlation with other indicators became weaker. The correlation between forest and grassland and water chemistry indicators increased in Aug.B and weakened with other land use patterns. In Jun.A, there was a significant positive correlation with SiO_2_ for cropland and forest and a significant negative correlation for impervious surfaces.

The subset of water chemistry parameters most correlated with bacterioplankton community composition included pH, DOC, NO₃-N, TN, and F⁻ (correlation coefficient = 0.735) in Jun.A (Table 2). This correlation strengthened significantly in Aug.B (correlation coefficient = 0.830), with SiO₂ and F⁻ constituting the best subset. DOC and F⁻ significantly influenced bacterioplankton communities before the rainy period. Subsequently, a broader set of parameters (DOC, NO₃-N, SiO₂, Ca²^+^, and F⁻) had a significant effect on both freshwater and cyanobacterial communities in Aug.B.

**TABLE 2:** Bioenv analysis finds the best subset of environmental variables^*a*^

Bacterioplankton community	Combination in Jun. A	Correlation	Combination in Aug. B	Correlation
Bacterioplankton	F^−^	0.565	F^−^	0.714
	DOC + TN	0.667	SiO_2_ + F^−^	0.830
	DOC + TN + F^−^	0.727	NO_3_-N + SiO_2_ + F^−^	0.828
	DOC + NH_4_ + TN + F^−^	0.734	DOC + SiO_2_ + Ca^2+^ + F^−^	0.818
	pH + DOC + N_O_3-N + TN + F^−^	0.735	DOC + SiO_2_ + Ca^2+^ + Mg^2+^ + F^−^	0.822
	DO + DOC + N_O_3-N + TN + F ^−^ + Cl^−^	0.732	DOC + NO_3_-N + SiO_2_ + Ca^2+^ + Mg^2+^ + F^−^	0.824
Freshwater bacterioplankton	F^−^	0.534	F^−^	0.702
	DOC + TN	0.682	SiO_2_ + F^−^	0.799
	DOC + TN + F^−^	0.725	NO_3_ + SiO_2_ + F^−^	0.804
	DOC + NH_4_ N + TN + F^−^	0.733	DOC + SiO_2_ + Ca^2+^ + F^−^	0.800
	DOC + NO_3_-N + NH_4_-N + TN + F^−^	0.722	DOC + SiO_2_ + Ca^2+^ + Mg^2+^ + F^−^	0.813
	DOC + NO_3_-N + NH_4_+ TN + F ^−^ + Cl^−^	0.720	DOC + NO_3_ N + SiO_2_ + Ca^2+^ + Mg^2+^ + F^−^	0.815
	pH + DOC + N_O_3-N + NH_4_-N + TN + F^−^ + Cl^−^	0.714	DOC + NO_3_-N + SiO_2_+ K^+^ +Ca^2+^ + Mg^2+^ + F^−^	0.803
Cyanobacteria	F^−^	0.436	SiO_2_	0.700
	DOC + F^−^	0.459	SiO_2_ + F^−^	0.764
	DOC + NO_3_-N F^−^	0.523	SiO_2_ + Na^+^ + F^−^	0.782
	DOC + NO_3_-N + F^−^ + Cl^−^	0.528	EC + NO_3_-N + SiO_2_ + F^−^	0.784
	pH + DOC + N_O_3-N + F^−^ + Cl^−^	0.538	EC + NO_3_-N + SiO_2_ + Ca^2+^ + F^−^	0.785
	pH + DOC + N_O_3-N + TN + F^−^ + Cl^−^	0.527	EC + DOC + N_O_3-N + SiO_2_ + Ca^2+^ + F^−^	0.790
	pH + DOC + N_O_3-N + NH_4_-N + TN + F^−^ + Cl^−^	0.516	EC + DOC + N_O_3-N + SiO_2_+ Ca^2+^ + F^−^ + Cl^−^	0.781
Bacterioplankton	Water	0.249	Forest	0.089
	Water + imperious surface	0.248	Forest + Grassland	0.120
	Forest + Water + imperious surface	0.246	Water + Forest + Grassland	0.118
Freshwater bacterioplankton	Water	0.256	Forest	0.089
	Water + imperious surface	0.251	Forest + Grassland	0.124
	Forest + Water + imperious surface	0.258	Water + Forest + Grassland	0.128
	Forest + Grassland + Water + imperious surface	0.198	Forest + Grassland + Water + imperious surface	0.123
Cyanobacteria	Water	0.253	Grassland	0.047
	Water + imperious surface	0.261	Forest + Grassland	0.089
	Forest + Water + imperious surface	0.234	Forest + Grassland + imperious surface	0.076

^*a*^ Environmental variables have the maximum rank correlation with bacterioplankton communities.

Since measured hydrological data were not available for every sampling point in the study area, we constructed numerical models to maximize the use of all sample data. Ten sampling sites (#2, #7, #13, #18, #23, #25, #27, #28, #30, #32) were measured, and data were obtained from the other 22 sampling sites using simulated data. Simulated water level results largely represent the actual water level change at each sampling site during the rainy season.

The simulated water levels showed reasonable agreement with measurements at the four hydrological stations although the results did not fully meet all accuracy criteria (Fig. S6). After validation with data from June to August 2022, the NSE values for three stations were above 0.6, indicating the model’s simulations were reasonable.

**Fig 7:**
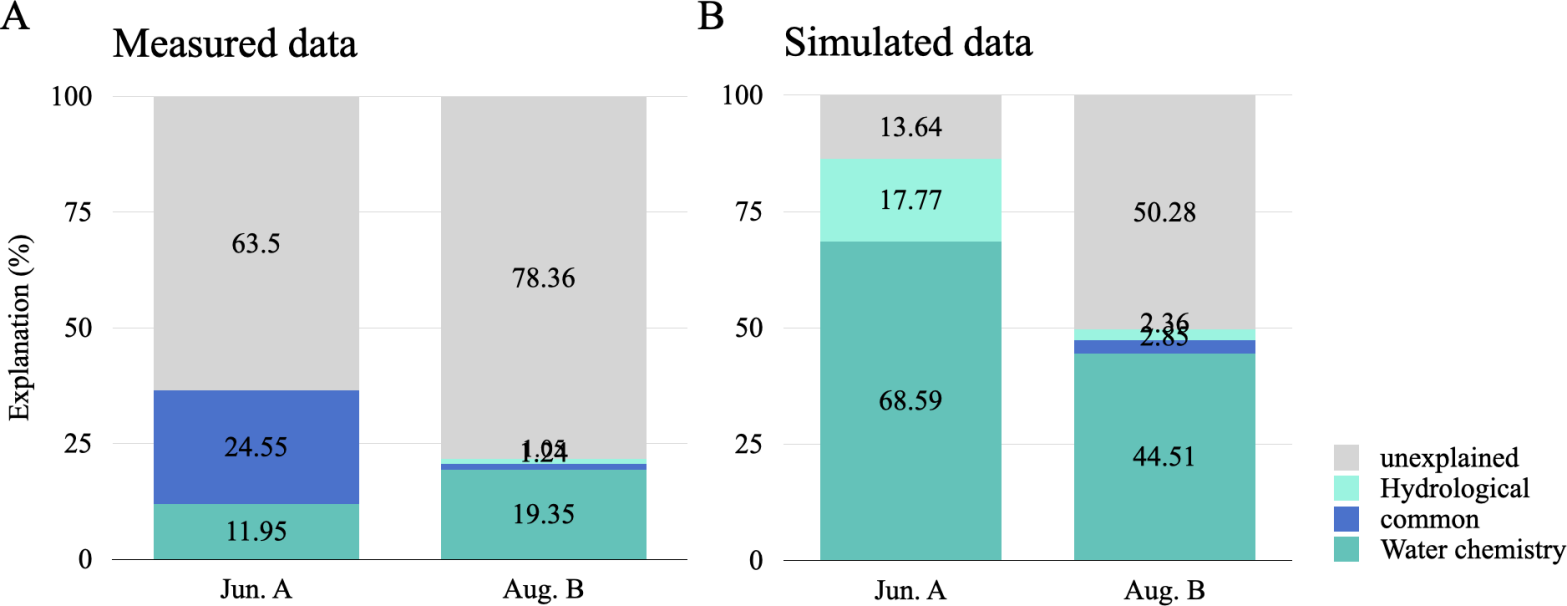
Variation portioning in cyanobacteria. Chemistry: selected from the subset of optimal water chemistry parameters in Table 2. Hydrology: using (**A**) measured and (**B**) simulated data.

Because the cyanobacterial community showed the correlation with hydrological characteristics of the river network (Fig. 6), this study aimed to understand the patterns of the cyanobacterial community response to environmental change processes (Fig. 7). Hydrological indicators included flow velocity and water level measured *in situ*, and water level included in the simulated data. The effects of the water chemistry were indicated using the subset of water chemical variables in Table 2 that had the greatest effect on the bacterioplankton community.

The unexplained fraction increased in Aug.B (post-plum rain) for both measured and simulated data, implying that the ability of hydrological and water chemistry variables to explain the distribution of cyanobacterial communities decreased in Aug.B (measured, A: 63.50%, B: 78.36%; simulated, A: 13.64%, B: 75.28%). Partially identical results were observed using measured and simulated data. The hydrological variables alone explain less than water chemistry. Differences in the results obtained using measured and modeled data, i.e., cyanobacterial communities, were more explained by the combination of simulated data.

## DISCUSSION

### Strong association of bacterioplankton communities before the plum rain

We found the connections of modules were weaker in the post-plum rain period and did not form a modular structure (Fig. 3 and Table 1). There were fewer edges and nodes, resulting in fewer connecting keystone species, showing a fragmented network and unstable community structure. Previous studies have found that as anthropogenic impacts increase, the complexity of the network structure decreases, the number of keystone species decreases, and the network modularity decreases (4, 48), i.e., anthropogenic activities can lead to a decrease in the modularity of the network. In this study, due to the high anthropogenic pressure on the plain river network itself, even though agricultural activities were reduced after the rainy period, a more connected network structure with reduced human activities was not observed, suggesting that changes in agricultural activities during the rainy period had a negligible effect on the bacterioplankton community. Although the modular structure in Aug.B was not as tightly connected as that in Jun.A, the keystone species still contributed to environmental stability. Luteolibacter, with the maximal stress and betweenness centrality, is involved in driving carbon and nitrogen biocycling processes in the water column (49). Limnobacter and f__Sphingomonadaceae are known for their ability to degrade a wide range of polycyclic aromatic hydrocarbons (PAHs) (50, 51). Curvibacter provides vitamins to cyanobacteria due to its genome possessing biosynthetic pathways for cobalamin (vitamin B12) and biotin (vitamin B7). This enabled Le et al. (52) to isolate this species during a cyanobacterial bloom, and together with Cyanobium PCC-6307, it became the cornerstone of the bacterial community in the river network after the rainy season.

### Drivers of bacterioplankton communities on anthropogenic pressure gradient

We found that rainfall increased the correlation between bacterioplankton communities and water chemistry, originating from more inputs from urban areas. FEAST analyses showed that rainfall brought species of unknown origin from urban areas compared to suburban and agricultural areas (Fig. 5). Less vegetation cover in urban areas relative to others promotes surface runoff, which is an important source of phosphorus (53). Hoke et al. (54) found that TP was significantly correlated with particle-associated samples after the rainy season, suggesting that phosphorus inputs from terrestrial runoff alleviated water phosphorus limitation. Rock-associated cations such as K^+^, Ca^2+^, Na^+^, and Mg^2+^ were significantly increased after the plum rain season (Fig. S9). SiO_2_ and F^−^ constitute the subset of water chemistry parameters most relevant to bacterioplankton (Table 2). F^−^ suggests exogenous inputs, with microorganisms mainly from urban areas affecting species interactions (4). Silica is only important to diatoms and forms the basis of their skeletal structure (53). At temperatures above 22°C, not only diatom blooms but also cyanobacteria are promoted (55). Community succession promotes algal cycling, and riverbed sediments convert particulate biochemical silica to SiO_2_ by mineralization and decomposition, which is an endogenous SiO_2_ production (53).

The inverse Simpson index was used to minimize the effect of rare taxa on diversity calculations, and it was found that the suburban river network provided a rich diversity of taxa, as evidenced by the significant increase in the inverse Simpson diversity index after the rainy season (Fig. 4), which suggests that the native species adapted to the environmental changes (56). We note that diversity was homogenized across land use types during the rainy season, suggesting that exogenous inputs from rainfall may have mitigated differences in native species abundance. McClary-Gutierrez et al. (57) similarly observed no difference in α-diversity between urban and agricultural areas, likely because rainfall during the wet season mixed pollution sources across land use types, leading to a convergence in community composition. They also found that freshwater bacteria were more aggregated in wider channels. Channel morphology (e.g., depth, width) and hydraulic geometry elements (e.g., flow velocity, bottom shear stress) may influence water quality by particle resuspension and dispersion (2, 58). The dilution effect of fertilizers and domestic and industrial wastewater in larger channels with higher discharge is stronger after the plum rain season, accelerating the movement of pollutants within the channel network and reducing the similarity of species, as evidenced by the fact that cyanobacteria are no longer aggregated and distributed in Aug.B (Fig. 6).

Hydrodynamics (e.g., flow-induced dilution) and water chemistry (e.g., nutrient availability) both influenced cyanobacteria abundance, but we did not observe a significantly stronger effect of hydrodynamics in either measured or simulated data (Fig. 7). Although studies have shown that hydrodynamic conditions can act directly on algal cells, affecting their growth and reproduction and intermediate competition, altering the water environment and nutrient status, suggesting that nutrients play a secondary role in determining algal community dynamics (59). Rainfall induces biotic (algal blooms) and abiotic (nutrients) upwelling, leading to community succession (60, 61). However, there are threshold values for the effect of hydrodynamic factors on algae, and the values vary for different algal species (62). When the hydraulic retention time is so long, water chemistry will become one of the main factors affecting algal proliferation (63). In this study, only the response of cyanobacteria to hydrodynamics was found (Fig. 6), and perhaps future studies targeting phytoplankton will yield further insights.

The variance partitioning analysis (VPA) revealed that a substantial proportion (75%–78% in Aug.B) of the variation in cyanobacterial community structures remained unexplained by the combined hydrological and water chemistry variables measured and simulated in this study (Fig. 7). This high unexplained variance is not uncommon in microbial ecology and underscores the complexity of forces shaping these communities. It strongly suggests the influence of other, unmeasured deterministic and stochastic factors. Several potential drivers could account for this “missing” variance. First, biotic interactions, such as competition, predation, and facilitation, likely play a crucial role. Our co-occurrence network analysis (Fig. 3 and Table 1) showed a fragmented post-rainy season structure with reduced complexity, indicating that such interactions are intense. The presence of keystone taxa like Luteolibacter (involved in nutrient cycling) and Curvibacter (which provides vitamins to cyanobacteria) suggests that microbially mediated metabolic interactions are a significant factor not captured by our environmental models. Second, we may have missed key environmental variables. The FEAST analysis (Fig. 5) indicated unexplained bacterial sources in urban areas post-rainfall, further hinting at the input of unquantified anthropogenic compounds (e.g., from urban runoff). Finally, stochastic processes, including ecological drift and random dispersal events during the high-flow rainy season, may contribute significantly, as evidenced by the homogenization of diversity across land-use types. Therefore, while our model successfully parameterized the known hydrological and chemical drivers, future studies aiming to achieve higher predictive power should integrate measures of biotic interactions, a broader suite of water quality parameters, and explicitly test the role of stochasticity.

Future studies could optimize the hydrodynamic model of the river network to understand cyanobacterial bloom outbreaks. In the studies of Li et al. (64) and Hui et al. (65), MIKE21 was used to simulate a river confluence (66) and implemented sediment simulation of a section of an urban river in MIKE21. The spatial scales modeled in the above studies were small, and the present study provides new insights into the study of bacterioplankton communities. Therefore, future research should develop coupled hydrodynamic-ecological models, integrating advanced computational techniques (e.g., machine learning for parameter calibration) to reduce uncertainty in simulating hydrodynamic effects on algal blooms and eutrophication in urban river networks.

### Caveats and limitations

This study provides insights into the bacterioplankton community within a plain river network; however, several limitations must be acknowledged. A primary limitation lies in its temporal scope, being based on a single sampling campaign conducted before and after the plum rain season in one year. While this design captures immediate hydrological shifts and their biological effects within a complete seasonal cycle, it inherently limits our ability to account for inter-annual variability. Factors such as varying rainfall intensity, frequency of extreme weather events, and long-term anthropogenic regulations may differ across years, potentially influencing the generalizability of our conclusions regarding community assembly mechanisms. Nevertheless, our integrated approach provides a robust snapshot and a reproducible framework. Future work will prioritize multi-year sampling to disentangle consistent, seasonally-driven processes from those driven by stochastic, inter-annual climatic fluctuations.

In parallel, it is also important to acknowledge the limitations of calibrating and validating the model using only one year of daily hydrological time series data, which impacts on the reliability of the model, particularly in regard to data accuracy. While some studies have utilized longer time series of data, such as daily 5-year (67), 20-year (32), and 30-year (5) series for simulation and validation on MIKE11, others have successfully employed shorter time periods of data for the purposes of modeling. To illustrate, Jahandideh-Tehrani et al. (68) utilized 1 year of hourly water level data to simulate less than 6 days of water level variations, and Panda et al. (69) employed 2 years of hourly water level data for the monsoon period only (four months) for model calibration and validation, respectively. Our approach, which utilized available data to its fullest potential for calibration, aligns with studies focused on establishing a foundational framework. However, extending the calibration period with multi-year data remains a critical future step to enhance predictive accuracy under complex and varying climatic scenarios.

A foremost limitation of our modeling approach is its inability to account for the complex anthropogenic regulation of urban waterways, particularly the operation of numerous smaller-scale hydraulic structures such as sluice gates and pumps. This simplification is a primary source of the noted discrepancy between simulated and observed data at highly engineered sites. A clear example is station Wuxi_4, located in the dense urban river network of Wuxi city. Here, impervious surfaces accelerate runoff, and water levels are constantly regulated—especially during the rainy season—to mitigate urban flooding and occasionally to prevent cyanobacterial invasion by closing sluices. This intensive management results in predominantly static and poor hydrodynamic conditions, as also documented by Xia et al. (70), which our model could not fully capture. Consequently, while the model reliably simulated the broader river network’s behavior (as evidenced by the good fit at the other three stations), its performance is limited in such intensely regulated urban environments. This underscores that our conclusions about factor dominance are most applicable to less anthropogenically perturbed systems. Future modeling efforts must prioritize the integration of these hydraulic infrastructures to accurately simulate urban river dynamics.

Furthermore, the MIKE11 HD model necessitates a smaller number of data sets for parameter calibration than other models. It is sufficient to set appropriate model parameters and input boundary conditions to represent the physical process of river flow. The objective of utilizing the MIKE11 model was to broaden the availability of hydrological data. The integration of additional data from gaging stations across expansive plain river networks has significantly fulfilled the requirements of modeling. In the present study, the available data were employed to their fullest potential, and the model was calibrated and validated to ensure its capability of representing the relationship between hydrodynamic changes and bacterioplankton within the existing conditions.

It is a limitation that the sampling was done only once before and after the rainy season in a cyclical pattern. Although the experimental design has some reasonable and practical considerations, the sampling period is limited to 2022, which is a shortcoming of the experimental design. Adding more years of sampling data will be considered in the future to improve the generalizability of the conclusions. Microbial communities are the result of long-term environmental selection and adaptation, and hydrologic variation, as one of the environmental factors to explain such high-dimensional data, has an inter-annual periodicity. The bacterioplankton community record in 1 rainy year fits a hydrological cycle pattern. Due to the seasonality of the hydrologic regime, only one sampling cycle provides the full picture of the riverine regime.

### Conclusion

Our analysis of the ecological network revealed that freshwater bacterioplankton—dominated by Proteobacteria, Actinobacteria, Bacteroidota, and Cyanobacteria (collectively > 90% of species)—exhibited significant spatial and temporal variability. Notably, suburban river networks retained higher biodiversity than urbanized areas, while the co-occurrence network’s complexity sharply declined after the rainy season, with keystone species diminishing from three to just one. These shifts were further quantified by marked reductions in Shannon and Chao1 indices, suggesting that hydrological changes during the rainy season critically reshape community structure.

By integrating hydrodynamic modeling (MIKE11) with field observations, we identified water chemistry as the dominant driver of bacterioplankton distribution, whereas physical factors had minimal impact on Cyanobacteria. Intriguingly, post-rainy season samples showed unexplained bacterioplankton sources predominantly in urban areas, hinting at anthropogenic influences. Despite minor discrepancies between measured and simulated data, our framework successfully parameterized spatial hydrological effects on microbial communities, offering a replicable approach to disentangle environmental and biological interactions. Moving forward, these findings provide a benchmark for refining models to predict ecological responses in regulated river networks under climate variability. Future work should aim to directly link hydrodynamic model outputs to microbial community assembly mechanisms, such as quantifying the relative roles of deterministic and stochastic processes during high-flow events.

## SUPPLEMENTAL MATERIAL

Supplemental material aem.01124-25-s0001.docx Supplemental methods; Fig. S1 to S9; Tables S1 and S2.

ASM does not own the copyrights to Supplemental Material that may be linked to, or accessed through, an article. The authors have granted ASM a non-exclusive, world-wide license to publish the Supplemental Material files. Please contact the corresponding author directly for reuse.

## References

[1] Hein T, Hauer C, Schmid M, Stöglehner G, Stumpp C, Ertl T, Graf W, Habersack H, Haidvogl G, Hood-Novotny R, et al.. 2021. The coupled socio-ecohydrological evolution of river systems: Towards an integrative perspective of river systems in the 21st century. Sci Total Environ 801:149619. doi:10.1016/j.scitotenv.2021.149619 34438150

[2] Pilla RM, Griffiths NA, Gu L, Kao S-C, McManamay R, Ricciuto DM, Shi X. 2022. Anthropogenically driven climate and landscape change effects on inland water carbon dynamics: What have we learned and where are we going? Glob Chang Biol 28:5601–5629. doi:10.1111/gcb.16324 35856254

[3] Ren L, Song S, Zhou Y. 2022. Evaluation of river ecological status in the plain river network area in the context of urbanization: a case study of 21 Rivers’ ecological status in Jiangsu Province, China. Ecol Indic 142:109172. doi:10.1016/j.ecolind.2022.109172

[4] Zhao J, Hein T, Yuan Q, Shu W, Huang X, Zhang X, Wang L. 2023. Co-occurrence patterns and assembly processes of abundant and rare bacterioplankton in plain river network areas of eastern China. Ecol Indic 150:110204. doi:10.1016/j.ecolind.2023.110204

[5] Karim F, Kinsey‐Henderson A, Wallace J, Godfrey P, Arthington AH, Pearson RG. 2014. Modelling hydrological connectivity of tropical floodplain wetlands via a combined natural and artificial stream network. Hydrol Process 28:5696–5710. doi:10.1002/hyp.10065

[6] Zhu Y, Zhou P, Gao S, Liu Y, Hu L. 2018. The study on water diversion and allocation of Subaiwei hydrographic net area in Changshu. IOP Conf Ser: Earth Environ Sci 189:022031. doi:10.1088/1755-1315/189/2/022031

[7] Wang Y, Gao C, Xu J, Zhang W, She L, Zhang Q, Bao R. 2022. Assessing the mechanism for flood control: a case of plain river network cities under extreme rainfalls. Environ Sci Pollut Res 30:38076–38098. doi:10.1007/s11356-022-24264-2 36576623

[8] Niu L, Li Y, Wang P, Zhang W, Wang C, Li J, Wu H. 2018. Development of a microbial community-based index of biotic integrity (MC-IBI) for the assessment of ecological status of rivers in the Taihu Basin, China. Ecol Indic 85:204–213. doi:10.1016/j.ecolind.2017.10.051

[9] Wu P, Qin B, Yu G, Deng J, Zhou J. 2016. Effects of nutrient on algae biomass during summer and winter in inflow rivers of Taihu Basin, China. Water Environ Res 88:665–672. doi:10.2175/106143016X14609975746767 27329063

[10] Wu Z, Wang X, Chen Y, Cai Y, Deng J. 2018. Assessing river water quality using water quality index in Lake Taihu Basin, China. Science of The Total Environment 612:914–922. doi:10.1016/j.scitotenv.2017.08.293 28886543

[11] Wu Z, Kong M, Cai Y, Wang X, Li K. 2019. Index of biotic integrity based on phytoplankton and water quality index: Do they have a similar pattern on water quality assessment? A study of rivers in Lake Taihu Basin, China. Science of The Total Environment 658:395–404. doi:10.1016/j.scitotenv.2018.12.216 30579197

[12] Marshall MM, Amos RN, Henrich VC, Rublee PA. 2008. Developing SSU rDNA metagenomic profiles of aquatic microbial communities for environmental assessments. Ecol Indic 8:442–453. doi:10.1016/j.ecolind.2007.04.007

[13] Wear EK, Koepfler ET, Smith EM. 2014. Spatiotemporal variability in dissolved organic matter composition is more strongly related to bacterioplankton community composition than to metabolic capability in a blackwater estuarine system. Estuaries Coast 37:119–133. doi:10.1007/s12237-013-9651-y

[14] Wu B, Wang P, Devlin AT, She Y, Zhao J, Xia Y, Huang Y, Chen L, Zhang H, Nie M, Ding M. 2022. Anthropogenic intensity-determined assembly and network stability of bacterioplankton communities in the Le’an River. Front Microbiol 13. doi:10.3389/fmicb.2022.806036 PMC9114710 35602050

[15] Zhou L, Wang S. 2023. The bright side of ecological stressors. Trends Ecol Evol 38:568–578. doi:10.1016/j.tree.2023.01.010 36906435

[16] Shu W, Zhang Q, Audet J, Li Z, Leng P, Qiao Y, Tian C, Chen G, Zhao J, Cheng H, Li F. 2024. Non-negligible N_2_O emission hotspots: Rivers impacted by ion-adsorption rare earth mining. Water Res 251:121124. doi:10.1016/j.watres.2024.121124 38237464

[17] Yan Z, Hao Z, Wu H, Jiang H, Yang M, Wang C. 2019. Co-occurrence patterns of the microbial community in polycyclic aromatic hydrocarbon-contaminated riverine sediments. J Hazard Mater 367:99–108. doi:10.1016/j.jhazmat.2018.12.071 30594728

[18] Gao Y, Zhang W, Li Y, Wu H, Yang N, Hui C. 2021. Dams shift microbial community assembly and imprint nitrogen transformation along the Yangtze River. Water Res 189:116579. doi:10.1016/j.watres.2020.116579 33160238

[19] Luo X, Xiang X, Huang G, Song X, Wang P, Fu K. 2019. Bacterial abundance and physicochemical characteristics of water and sediment associated with hydroelectric dam on the Lancang River China. IJERPH 16:2031. doi:10.3390/ijerph16112031 31181632 PMC6603985

[20] Banerjee S, Schlaeppi K, van der Heijden MGA. 2018. Keystone taxa as drivers of microbiome structure and functioning. Nat Rev Microbiol 16:567–576. doi:10.1038/s41579-018-0024-1 29789680

[21] Olesen JM, Bascompte J, Dupont YL, Jordano P. 2007. The modularity of pollination networks. Proc Natl Acad Sci U S A 104:19891–19896. doi:10.1073/pnas.0706375104 18056808 PMC2148393

[22] Jones AC, Hambright KD, Caron DA. 2018. Ecological patterns among bacteria and microbial eukaryotes derived from network analyses in a low-salinity lake. Microb Ecol 75:917–929. doi:10.1007/s00248-017-1087-7 29110066

[23] Zhang T, Xu S, Yan R, Wang R, Gao Y, Kong M, Yi Q, Zhang Y. 2022. Similar geographic patterns but distinct assembly processes of abundant and rare bacterioplankton communities in river networks of the Taihu Basin. Water Res 211:118057. doi:10.1016/j.watres.2022.118057 35066261

[24] Broderick CM, Benucci GMN, Bachega LR, Miller GD, Evans SE, Hawkes CV. 2025. Long-term climate establishes functional legacies by altering microbial traits. ISME J 19:wraf005. doi:10.1093/ismejo/wraf005 39804671 PMC11805608

[25] Das AK, Lee D-S, Woo Y-J, Sultana S, Mahmud A, Yun B-W. 2025. The impact of flooding on soil microbial communities and their functions: a review. Stresses 5:30. doi:10.3390/stresses5020030

[26] Kun S, Boczonádi I, Nagy PT, Szabó A, Tóth FA, Fehér ZZ, Magyar T, Madar LA, Szűcs I, Tamás J, Nagy A. 2025. Impact of intense rainfall event on the physicochemical and microbiological characteristics of an urban stream, Vienna, Austria

[27] Liang Y, Liao W, Wang H. 2025. Efficient urban flooding management: a multi-physical-process-oriented flood modelling and analysis method. Sustainability 17:1124. doi:10.3390/su17031124

[28] Lu M, Kang C, Yu Z, Zhang X. 2023. Coordination of flood control under urbanization on the Taihu Plain: Basin, City and Region Perspectives. Water (Basel) 15:3723. doi:10.3390/w15213723

[29] Gao X, Liu Y, Gao C, Qing D, Wang Q, Cai Y. 2024. Coupling and comparison of physical mechanism and machine learning models for water level simulation in plain river network area. Appl Sci (Basel) 14:12008. doi:10.3390/app142412008

[30] Tang H, Yuan S, Cao H. 2023. Theory and practice of hydrodynamic reconstruction in plain river networks. Engineering (Beijing) 24:202–211. doi:10.1016/j.eng.2022.01.015

[31] Xiang L, Ma M, Jiao L, Liao Z. 2020. Water diversion and allocation for typical confined polder river-net in Taihu Basin. Water Policy 22:561–573. doi:10.2166/wp.2020.107

[32] Wang Y, Qiu R, Tao Y, Wu J. 2023. Influence of the impoundment of the three gorges reservoir on hydrothermal conditions for fish habitat in the Yangtze River. Environ Sci Pollut Res 30:10995–11011. doi:10.1007/s11356-022-22930-z 36087184

[33] Schuchert P, Kregting L, Pritchard D, Savidge G, Elsäßer B. 2018. Using coupled hydrodynamic biogeochemical models to predict the effects of tidal turbine arrays on phytoplankton dynamics. JMSE 6:58. doi:10.3390/jmse6020058

[34] Li Y, Liao Z, Hui C, Zheng J, Yuan S, Zhang W. 2023. Hydraulic characteristics in channel confluence affect the nitrogen dynamics through altering interactions among multi-trophic microbiota. Water Res 235:119882. doi:10.1016/j.watres.2023.119882 36947927

[35] Carney R, Mitrovic S, Jeffries T, Westhorpe D, Curlevski N, Seymour J. 2015. River bacterioplankton community responses to a high inflow event. Aquat Microb Ecol 75:187–205. doi:10.3354/ame01758

[36] Wilson AM, Martin SL, Verhougstraete MP, Kendall AD, Zimmer-Faust AG, Rose JB, Bell ML, Hyndman DW. 2022. Detangling seasonal relationships of fecal contamination sources and correlates with indicators in michigan watersheds. Microbiol Spectr 10:e0041522. doi:10.1128/spectrum.00415-22 35730960 PMC9431008

[37] Xie Z, Chen S, Huang J, Li D, Lu X. 2023. Patterns and drivers of fecal coliform exports in a typhoon-affected watershed: insights from 10-year observations and SWAT model. J Clean Prod 406:137044. doi:10.1016/j.jclepro.2023.137044

[38] Deng X. 2019. Correlations between water quality and the structure and connectivity of the river network in the Southern Jiangsu Plain, Eastern China. Sci Total Environ 664:583–594. doi:10.1016/j.scitotenv.2019.02.048 30763839

[39] Callahan BJ, McMurdie PJ, Rosen MJ, Han AW, Johnson AJA, Holmes SP. 2016. DADA2: High-resolution sample inference from Illumina amplicon data. Nat Methods 13:581–583. doi:10.1038/nmeth.3869 27214047 PMC4927377

[40] Newton RJ, Jones SE, Eiler A, McMahon KD, Bertilsson S. 2011. A guide to the natural history of freshwater lake bacteria. Microbiol Mol Biol Rev 75:14–49. doi:10.1128/MMBR.00028-10 21372319 PMC3063352

[41] Rohwer RR, Hamilton JJ, Newton RJ, McMahon KD. 2018. TaxAss: leveraging a custom freshwater database achieves fine-scale taxonomic resolution. mSphere 3:00327–18. doi:10.1128/mSphere.00327-18 PMC6126143 30185512

[42] Hsieh TC, Ma KH, Chao A. 2016. iNEXT: an R package for rarefaction and extrapolation of species diversity ( H ill numbers). Methods Ecol Evol 7:1451–1456. doi:10.1111/2041-210X.12613

[43] Liaw A, Wiener M. 2002. Classification and regression by randomForest

[44] Bastian M, Heymann S, Jacomy M. 2009. Gephi: an open source software for exploring and manipulating networks. ICWSM 3:361–362. doi:10.1609/icwsm.v3i1.13937

[45] Shenhav L, Thompson M, Joseph TA, Briscoe L, Furman O, Bogumil D, Mizrahi I, Pe’er I, Halperin E. 2019. FEAST: fast expectation-maximization for microbial source tracking. Nat Methods 16:627–632. doi:10.1038/s41592-019-0431-x 31182859 PMC8535041

[46] Oksanen J. 2012. Constrained ordination: tutorial with R and vegan

[47] Borcard D, Legendre P, Drapeau P. 1992. Partialling out the spatial component of ecological variation. Ecology 73:1045–1055. doi:10.2307/1940179

[48] Kraemer SA, Barbosa da Costa N, Shapiro BJ, Fradette M, Huot Y, Walsh DA. 2020. A large-scale assessment of lakes reveals a pervasive signal of land use on bacterial communities. ISME J 14:3011–3023. doi:10.1038/s41396-020-0733-0 32770118 PMC7784917

[49] Zhang L, Yin W, Wang C, Zhang A, Zhang H, Zhang T, Ju F. 2021. Untangling microbiota diversity and assembly patterns in the world’s largest water diversion canal. Water Res 204:117617. doi:10.1016/j.watres.2021.117617 34555587

[50] Chen Y, Feng X, He Y, Wang F. 2016. Genome analysis of a Limnobacter sp. identified in an anaerobic methane-consuming cell consortium. Front Mar Sci 3. doi:10.3389/fmars.2016.00257

[51] Leys N, Ryngaert A, Bastiaens L, Verstraete W, Top EM, Springael D. 2004. Occurrence and phylogenetic diversity of Sphingomonas strains in soils contaminated with polycyclic aromatic hydrocarbons. Appl Environ Microbiol 70:1944–1955. doi:10.1128/AEM.70.4.1944-1955.2004 15066784 PMC383131

[52] Le VV, Ko S-R. 2023. Comparative genome analysis of the genus Curvibacter and the description of Curvibacter microcysteis sp. nov. and Curvibacter cyanobacteriorum sp. nov., isolated from fresh water during the cyanobacterial bloom period. J Microbiol Biotechnol:1428–1436. doi:10.4014/jmb.2306.06017 37644736 PMC10699270

[53] Ji Z. 2017. Hydrodynamics and water quality: modeling rivers, lakes, and estuaries. John Wiley & Sons.

[54] Hoke A, Woodhouse J, Zoccarato L, McCarthy V, de Eyto E, Calderó-Pascual M, Geffroy E, Dillane M, Grossart H-P, Jennings E. 2020. Impacts of Extreme weather events on bacterial community composition of a temperate humic lake. Water (Basel) 12:2757. doi:10.3390/w12102757

[55] Mikhailov IS, Galachyants YP, Bukin YS, Petrova DP, Bashenkhaeva MV, Sakirko MV, Blinov VV, Titova LA, Zakharova YR, Likhoshway YV. 2022. Seasonal succession and coherence among bacteria and microeukaryotes in lake Baikal. Microb Ecol 84:404–422. doi:10.1007/s00248-021-01860-2 34510242

[56] Jousset A, Bienhold C, Chatzinotas A, Gallien L, Gobet A, Kurm V, Küsel K, Rillig MC, Rivett DW, Salles JF, van der Heijden MGA, Youssef NH, Zhang X, Wei Z, Hol WHG. 2017. Where less may be more: how the rare biosphere pulls ecosystems strings. ISME J 11:853–862. doi:10.1038/ismej.2016.174 28072420 PMC5364357

[57] McClary-Gutierrez JS, Driscoll Z, Nenn C, Newton RJ. 2021. Human fecal contamination corresponds to changes in the freshwater bacterial communities of a large river basin. Microbiol Spectr 9:e0120021. doi:10.1128/Spectrum.01200-21 34494860 PMC8557911

[58] Zhou L, Liu L, Chen W-Y, Sun J-J, Hou S-W, Kuang T-X, Wang W-X, Huang X-D. 2020. Stochastic determination of the spatial variation of potentially pathogenic bacteria communities in a large subtropical river. Environ Pollut 264:114683. doi:10.1016/j.envpol.2020.114683 32388300

[59] Padisák J. 1997. Cylindrospermopsis raciborskii (Woloszynska) Seenayya et Subba Raju, an expanding, highly adaptive cyanobacterium: worldwide distribution and review of its ecology. Arch FÜR Hydrobiol Suppl Monogr BEITRAGE 107:4.

[60] Fawcett SE, Ward BB. 2011. Phytoplankton succession and nitrogen utilization during the development of an upwelling bloom. Mar Ecol Prog Ser 428:13–31. doi:10.3354/meps09070

[61] Teeling H, Fuchs BM, Becher D, Klockow C, Gardebrecht A, Bennke CM, Kassabgy M, Huang S, Mann AJ, Waldmann J, et al.. 2012. Substrate-controlled succession of marine bacterioplankton populations induced by a phytoplankton bloom. Science 336:608–611. doi:10.1126/science.1218344 22556258

[62] Liang P, Wang H, Ma F. 2013. Effect of hydrodynamic conditions on water eutrophication: a review. J Lake Sci 8. doi:10.18307/2013.0401

[63] Ren J, Zhu G, Jin Y, Xu H, Zhu M, Xia M, Yu L, Li H, Zhang Y, Qin B. 2017. Combined effects of water exchange rate and nutrient on diatom proliferation in Hengshan Reservoir, Taihu Basin. J Lake Sci 29:604–616. doi:10.18307/2017.0309

[64] Li P, Li W, Dumbrell AJ, Liu M, Li G, Wu M, Jiang C, Li Z. 2020. Spatial variation in soil fungal communities across paddy fields in subtropical China. mSystems 5:e00704–19. doi:10.1128/msystems.00704-19 31911465 PMC6946795

[65] Hui C, Li Y, Zhang W, Yang G, Wang H, Gao Y, Niu L, Wang L, Zhang H. 2021. Coupling genomics and hydraulic information to predict the nitrogen dynamics in a channel confluence. Environ Sci Technol 55:4616–4628. doi:10.1021/acs.est.0c04018 33760605

[66] Zhang W, Wang H, Li Y, Lin L, Hui C, Gao Y, Niu L, Zhang H, Wang L, Wang P, Wang C. 2020. Bend-induced sediment redistribution regulates deterministic processes and stimulates microbial nitrogen removal in coarse sediment regions of river. Water Res 170:115315. doi:10.1016/j.watres.2019.115315 31778969

[67] Teshome FT, Bayabil HK, Thakural LN, Welidehanna FG. 2020. Verification of the MIKE11-NAM Model for Simulating Streamflow. JEP 11:152–167. doi:10.4236/jep.2020.112010

[68] Jahandideh-Tehrani M, Helfer F, Zhang H, Jenkins G, Yu Y. 2020. Hydrodynamic modelling of a flood-prone tidal river using the 1D model MIKE HYDRO River: calibration and sensitivity analysis. Environ Monit Assess 192:97. doi:10.1007/s10661-019-8049-0 31912301

[69] Panda RK, Pramanik N, Bala B. 2010. Simulation of river stage using artificial neural network and MIKE 11 hydrodynamic model. Computers & Geosciences 36:735–745. doi:10.1016/j.cageo.2009.07.012

[70] Xia Y, Wang H, He X, Yuan W, Zeng Y, Yan H, Zhang X, Tu Y. 2021. Spatiotemporal heterogeneity of hydrodynamic forces and water quality in typical lakeside river networks in Taihu Basin (in Chinese). J Lake Sci 4. doi:10.18307/2021.0412

