# Supplementary material for: Changes and drivers of bacterioplankton communities within plain river networks during the rainy season (high inflow event): simulation of the water level using the MIKE11 model
Source: Appl Environ Microbiol. 2025 Nov 28;91(12):e01124-25. doi: 10.1128/aem.01124-25 (PMC12724245; doi:10.1128/aem.01124-25)
Supplement: Supplemental material — Supplemental methods; Fig. S1 to S9; Tables S1 and S2. [file aem.01124-25-s0001.docx]

**Supplementary Material**

**Brief Caption**

**1 Material and method**

1.1 Molecular analysis

1.2 Hydrological modeling and integration with ecological data

**Reference**

**2 Supplementary Figures**

**FIG S1** Conceptual framework of the study, showing the investigation of bacterioplankton communities along an anthropogenic gradient and the application of the MIKE11 model.

**FIG S2** Daily precipitation (blue bar; mm/d; right y-axis) and air temperature (grey line; mm; left y-axis) in the study area from Jan. 1st to Dec. 31st, 2022. The pale blue shaded area indicates the period of plum rain during the sampling time. *Source: Taihu Basin Authority of Ministry of water resources; **Source: National precipitation criterion of China (GB/T 28592–2012).

**FIG S3** Three groups of 32 sampling sites were categorized according to the proportion of land use types in the surrounding 1,000-meter area.

**FIG S4** Generalized map of river network in MIKE11 model.

**FIG S5** Measurement and simulation of water level at hydrological stations from February 7 to March 11, 2022 (Calibration periods).

**FIG S6** Measurement and simulation of water level at hydrological stations from June 13 to August 26, 2022 (Validation periods).

**FIG S7** Rarefaction curve based on observed species (Sobs) before (Jun. A) and after (Aug. B) the plum rain season.

**FIG S8** Composition and distributions of bacterioplankton communities before (Jun. A) and after (Aug. B) the Plum rainy season. (A) Composition of bacterioplankton at phylum level, (B) freshwater bacterioplankton, and (C) cyanobacterial communities.

**FIG S9** Scatterplot showing the content of water chemistry variables before (Jun. A) and after (Aug. B) the Plum Rain season. ****p* < 0.001, ***p* < 0.01, **p* < 0.05.

**3 Supplementary Tables**

**Table S1** Information on the top five keystone species in before and after the rainy season.

**Table S2** Difference of correlation between land use types and water chemistry before and after the Plum rainy season.

# Material and method

## Molecular analysis

Samples were subjected to three DNA extractions using the E.Z.N.A.^®^ Soil DNA Kit (Omega Bio-Tek, Unite States) according to the manufacturer's protocol. The quality of the obtained DNA samples was checked using a NanoDrop 2000 spectrophotometer (Thermo Fisher Scientific, Unite States). The extracted DNA samples were amplified by forward primer 338F (5’-ACTCCTACGG-AGGCAGCAG-3’) and reverse primer 806R (5’-GGACTACHVGGTWTCTAAT-3’) to amplify the V3-V4 gene fragment of 16S rDNA, i.e., Polymerase Chain Reaction (PCR) (PCR, ABI GeneAmp^®^ 9700, Unite States). The amplification procedure was as follows: pre-denaturation at 95°C for 3 min, 27 cycles (denaturation at 95°C for 30 s, annealing at 55°C for 30 s, and extension at 72°C for 45 s), followed by a stable extension at 72°C for 10 min, and then stopping after cooling down to 10°C, and finally, storage at 4°C. The PCR reaction system was as follows: 5× TransStart FastPfu buffer, 4 μL; 2.5 mM triple Deoxynucleoside triphosphate (dNTPs) 2 μL; forward primer (5 μM) 0.8 μL, reverse primer (5 μM) 0.8 μL, TransStart FastPfu DNA polymerase, 0.4 μL; 10 ng of template DNA, made up to 20 μL with double-distilled water (ddH_2_O). Three replicates were performed for each sample.

PCR products from the same samples were mixed and the PCR products were recovered using a 2% agarose gel. The gel was cut using the AxyPrep DNA Gel Extraction Kit (Axygen Biosciences, United States). The recovered products were purified, eluted by Tris_HCl, 2% agarose gel electrophoresis, and the recovered products were detected and quantified by Quantus™ Fluorometer (Promega, United States) blue fluorescence quantification system.

The library was constructed using NEXTflex^TM^ Rapid DNA-Seq Kit (BiooScientific, United States). MiSeq sequencing has paired-end sequence data, i.e., after obtaining PE reads for sample splitting, quality control and filtering of double-ended Reads were performed according to the sequencing quality, and at the same time, splicing was performed according to the overlap relationship between double-ended Reads to obtain the optimized data after quality control splicing. The data of each sample were distinguished according to the barcode sequence, and the extracted PE data of the original sequence were saved in fastq format. fq1 and fq2 files were available for each sample of PE data, which were the Reads sequences of the two ends of sequencing in order and one-to-one correspondence. Sequences were merged and quality filtered for denoising by fastp^[[1]](#footnote-1)^ (v. 0.19.6) and FLASH^[[2]](#footnote-2)^ (v. 1.2.11) software (1, 2).

In a sample of the given order q (q = 0,1,2), the effect of rare species will become smaller as q increases.

$$\begin{aligned} {}^{q}D={(\sum_{i=1}^{S} p_{i}^{q})}^{\frac{1}{1-q}}\#\ldots\ldots\left( 1 \right) \end{aligned}$$

Where, S is the number of species in a sample; P_i_ is the relative abundance of the ith species.

## Hydrological modeling and integration with ecological data

The MIKE11 HD model uses Abbott's six-point implicit difference scheme to solve the Saint-Venant equations, as follows:

$$\begin{aligned} \frac{\partial Q}{\partial x}+b\frac{\partial A}{\partial t}=q\#\ldots\ldots\left( 2 \right) \end{aligned}$$

$$\begin{aligned} \frac{\partial Q}{\partial t}+\frac{\partial\left( \alpha\frac{Q^{2}}{A} \right)}{\partial x}+gA\frac{\partial h}{\partial x}+\frac{gQ\left| Q \right|}{C^{2}AR}=0\#\ldots\ldots\left( 3 \right) \end{aligned}$$

Where, Q is cross section flow, m^3^/s; x is spatial coordinates, m; A is cross section area, m^2^; Q is side into the flow, m^3^/s; t is time coordinates, s; 𝛼 is momentum distribution coefficient, m/s^2^; g is acceleration of gravity, m/s^2^; h is water level, m; C is Chezy coefficient; R is hydraulic radius/resistance, m.

# Reference

1. Chen S, Zhou Y, Chen Y, Gu J. 2018. fastp: an ultra-fast all-in-one FASTQ preprocessor. Bioinformatics 34:i884–i890.

2. Magoč T, Salzberg SL. 2011. FLASH: fast length adjustment of short reads to improve genome assemblies. Bioinformatics 27:2957–2963.

# Supplementary Figures

1.
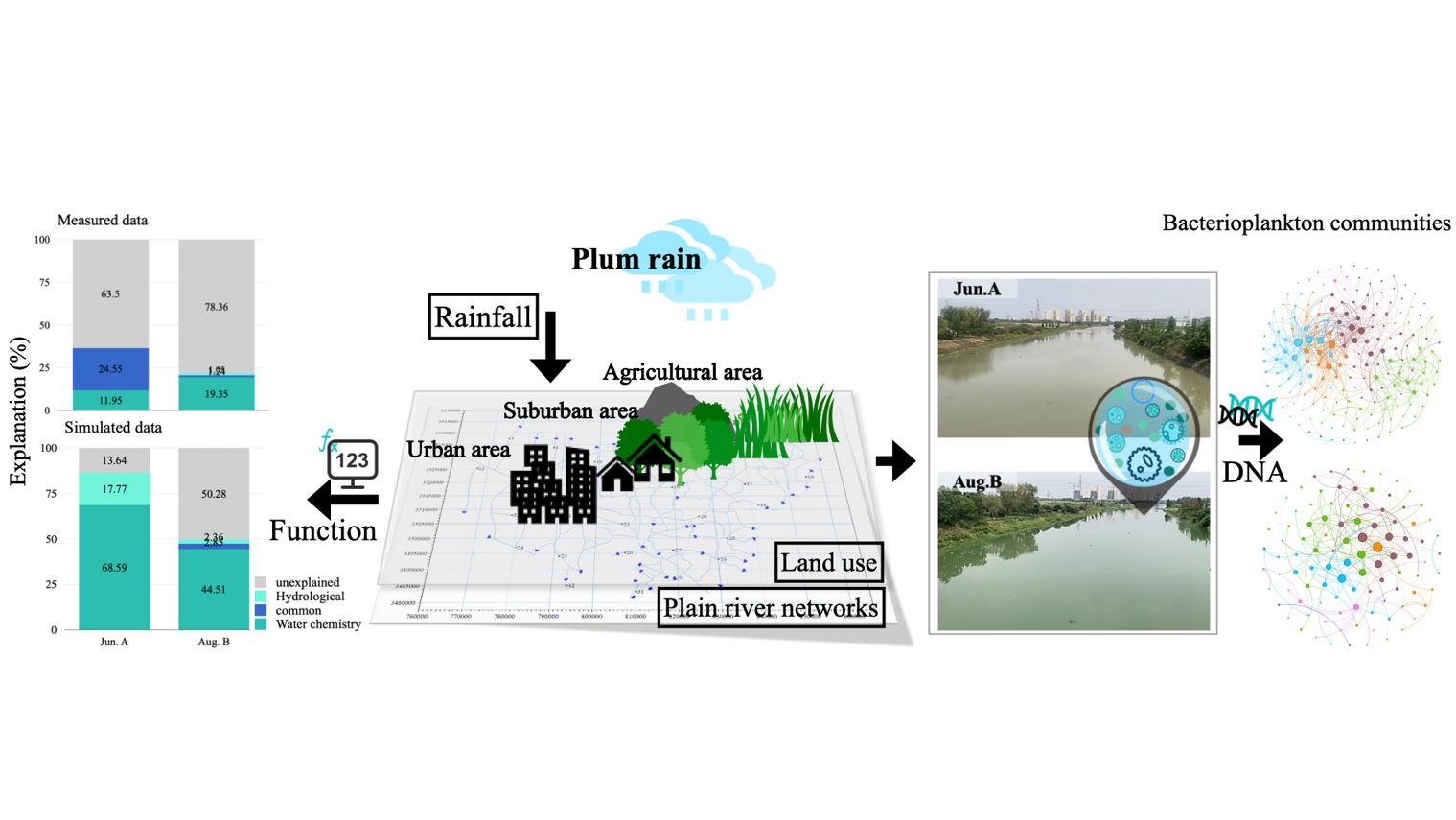


**FIG S1** Conceptual framework of the study, showing the investigation of bacterioplankton communities along an anthropogenic gradient and the application of the MIKE11 model.


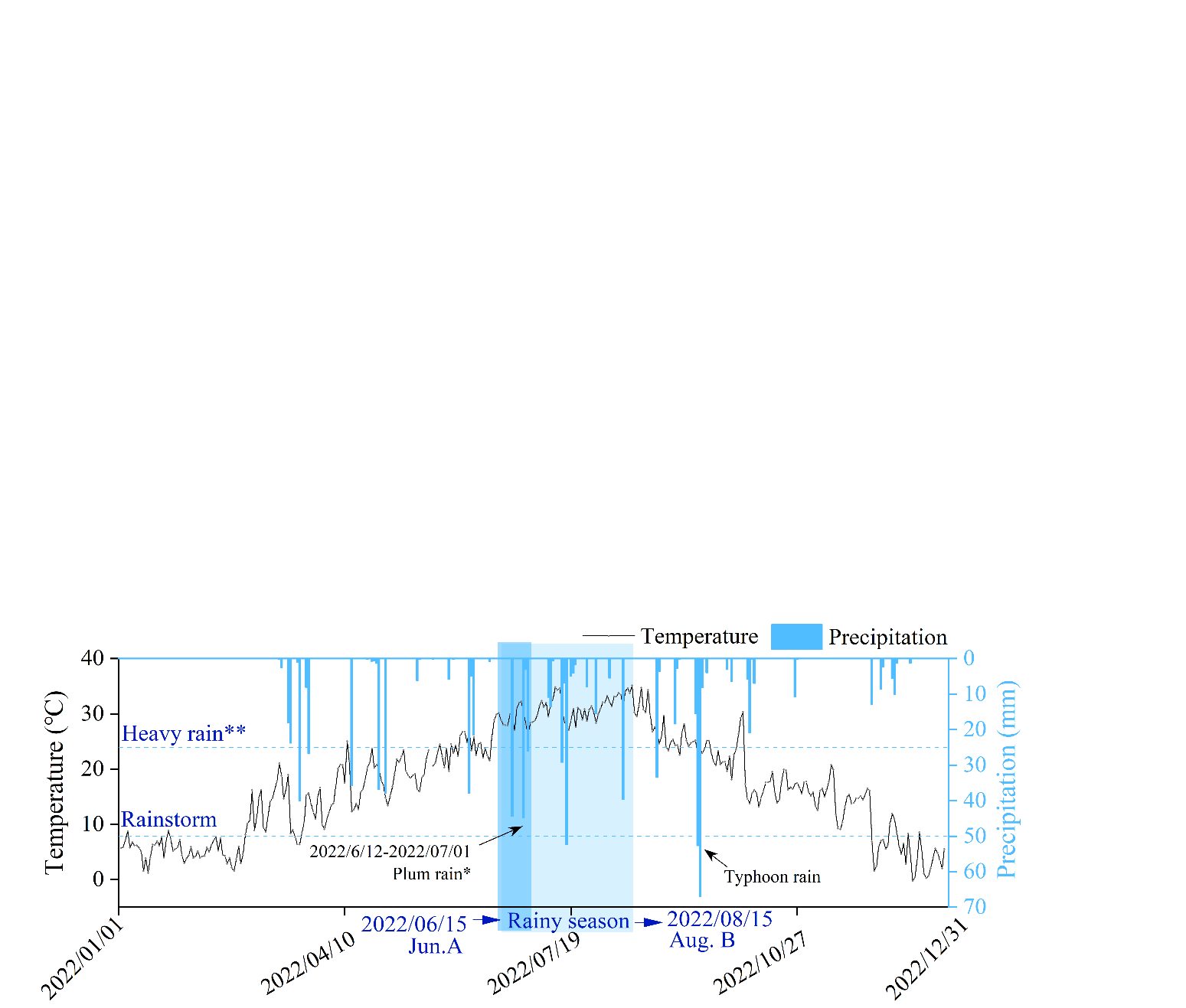


FIG S2 Daily precipitation (blue bar; mm/d; right y-axis) and air temperature (grey line; mm; left y-axis) in the study area from Jan. 1^st^ to Dec. 31^st^, 2022. The pale blue shaded area indicates the period of plum rain during the sampling time. *Source: Taihu Basin Authority of Ministry of water resources^[[3]](#footnote-3)^; **Source: National precipitation criterion of China (GB/T 28592–2012).


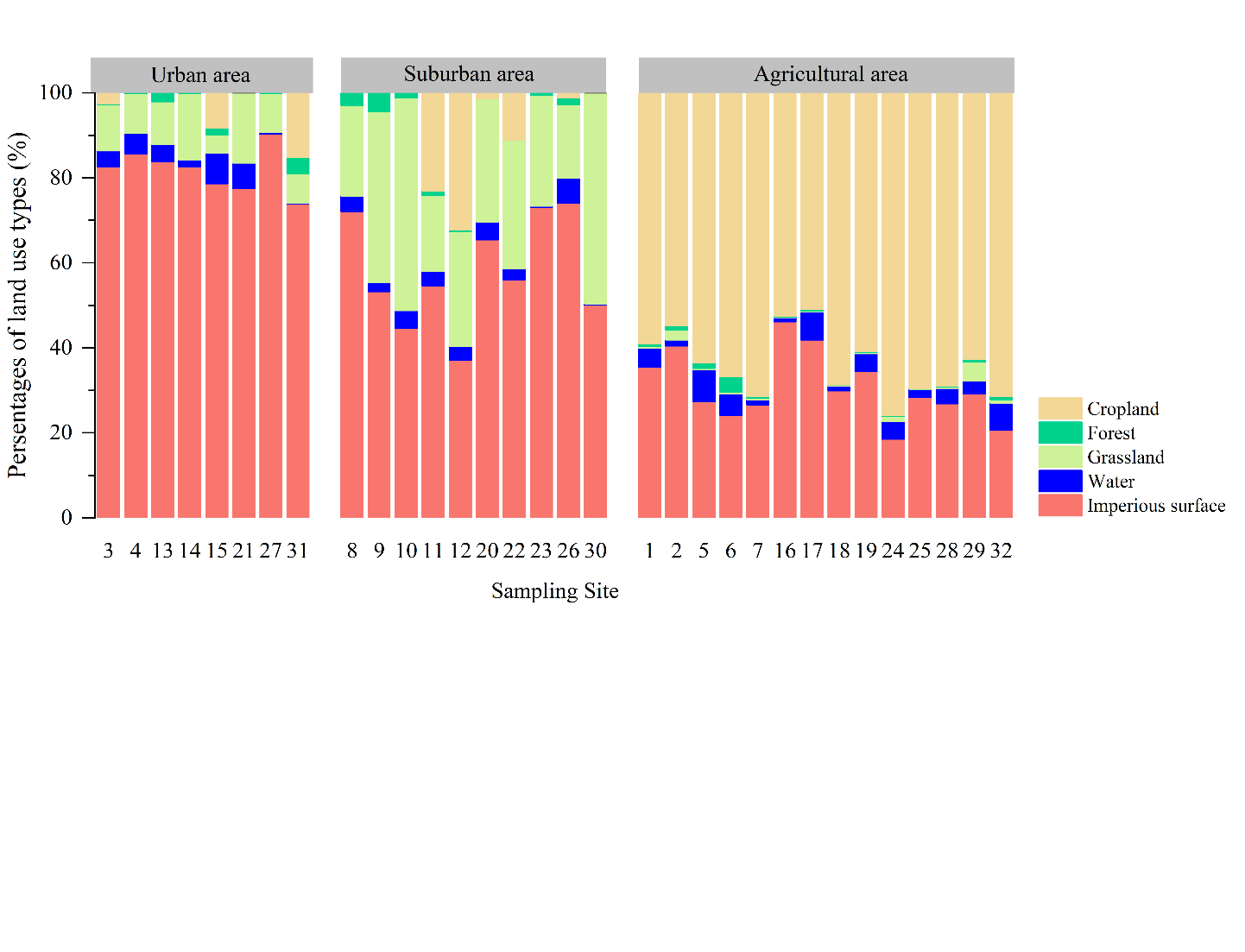


FIG S3 Three groups of 32 sampling sites were categorized according to the proportion of land use types in the surrounding 1,000-meter area.


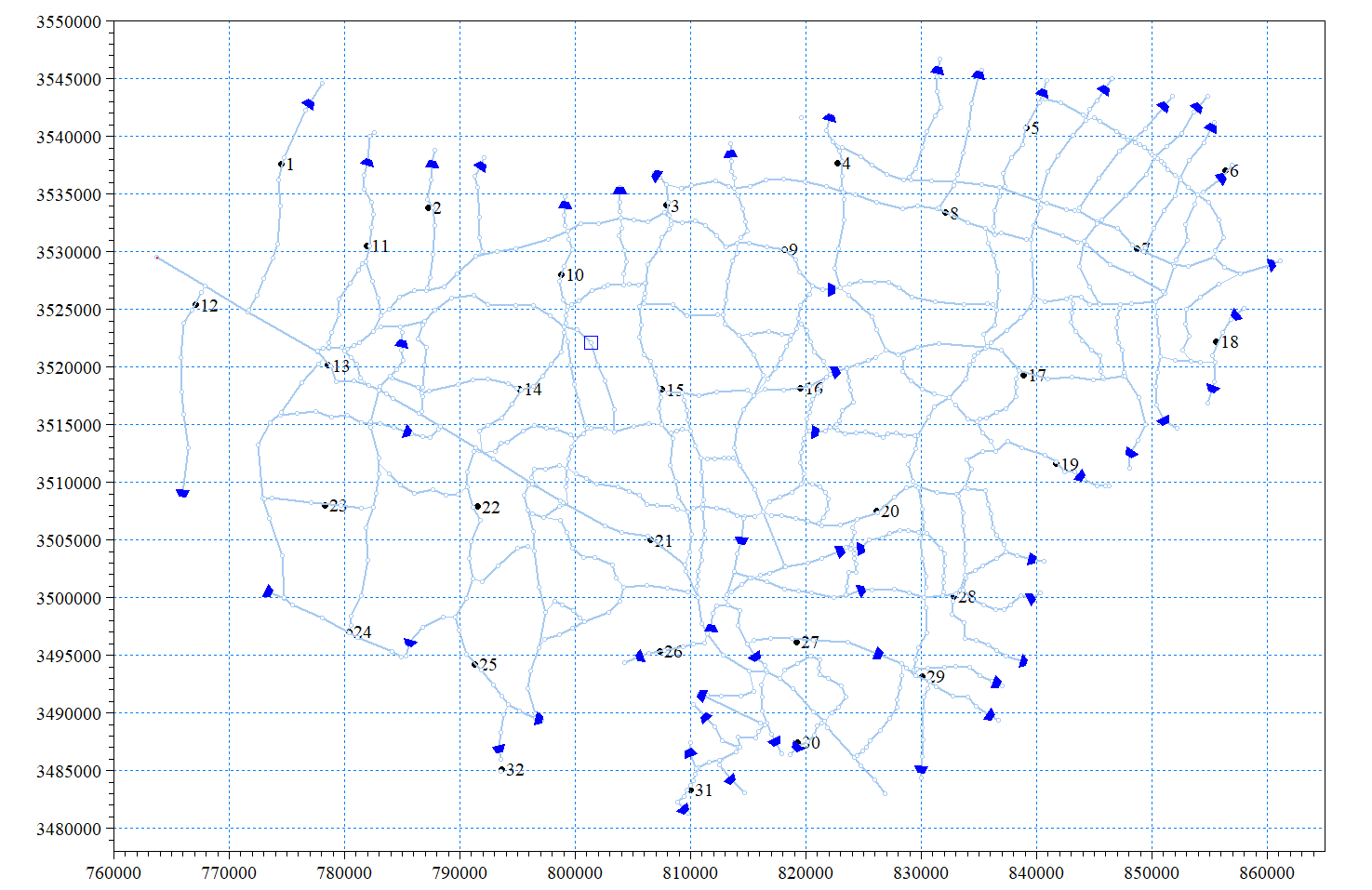


FIG S4 Generalized map of river network in MIKE11 model.


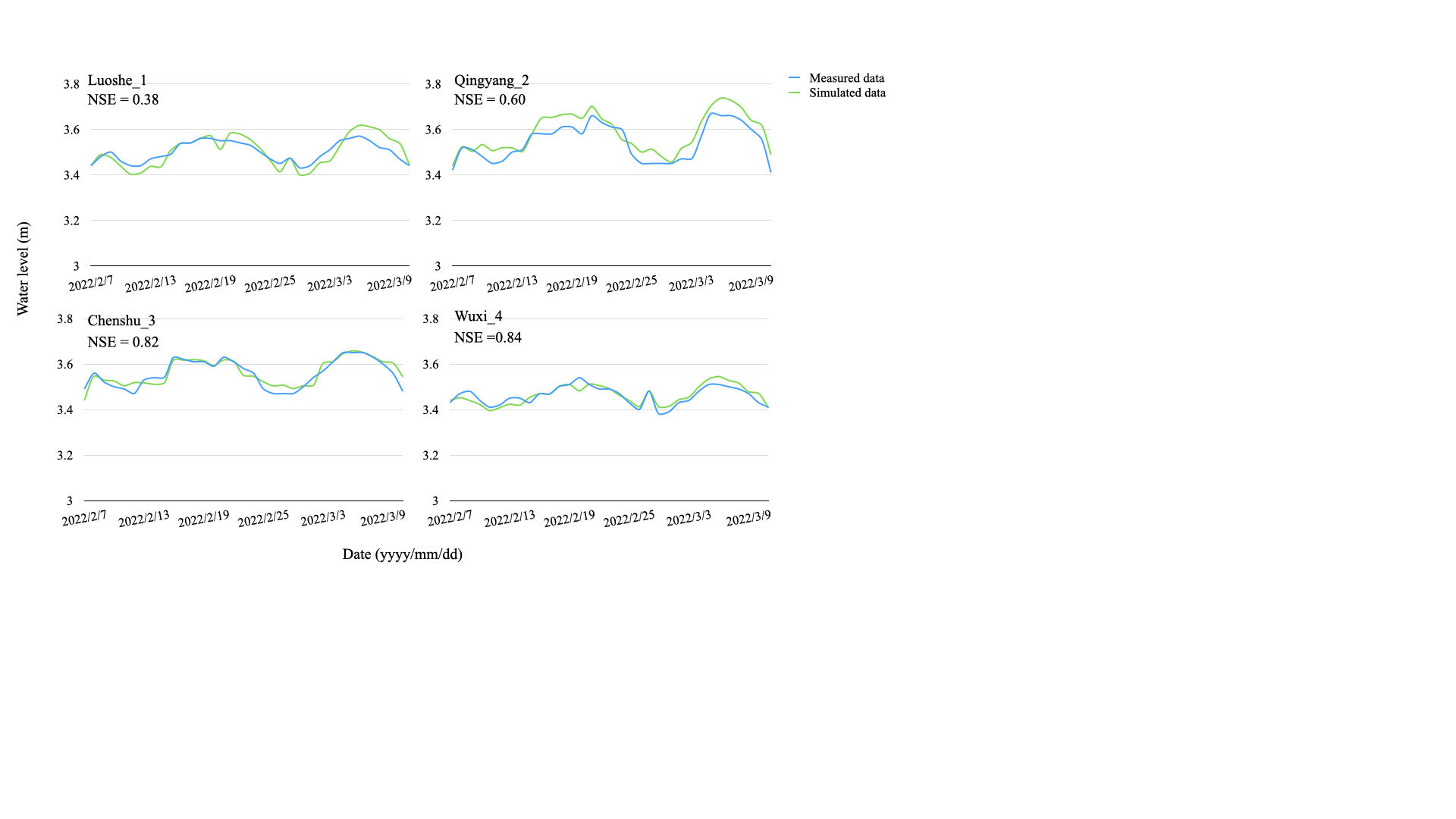


**FIG S5** Measurement and simulation of water level at hydrological stations from February 7 to March 11, 2022 (Calibration periods).


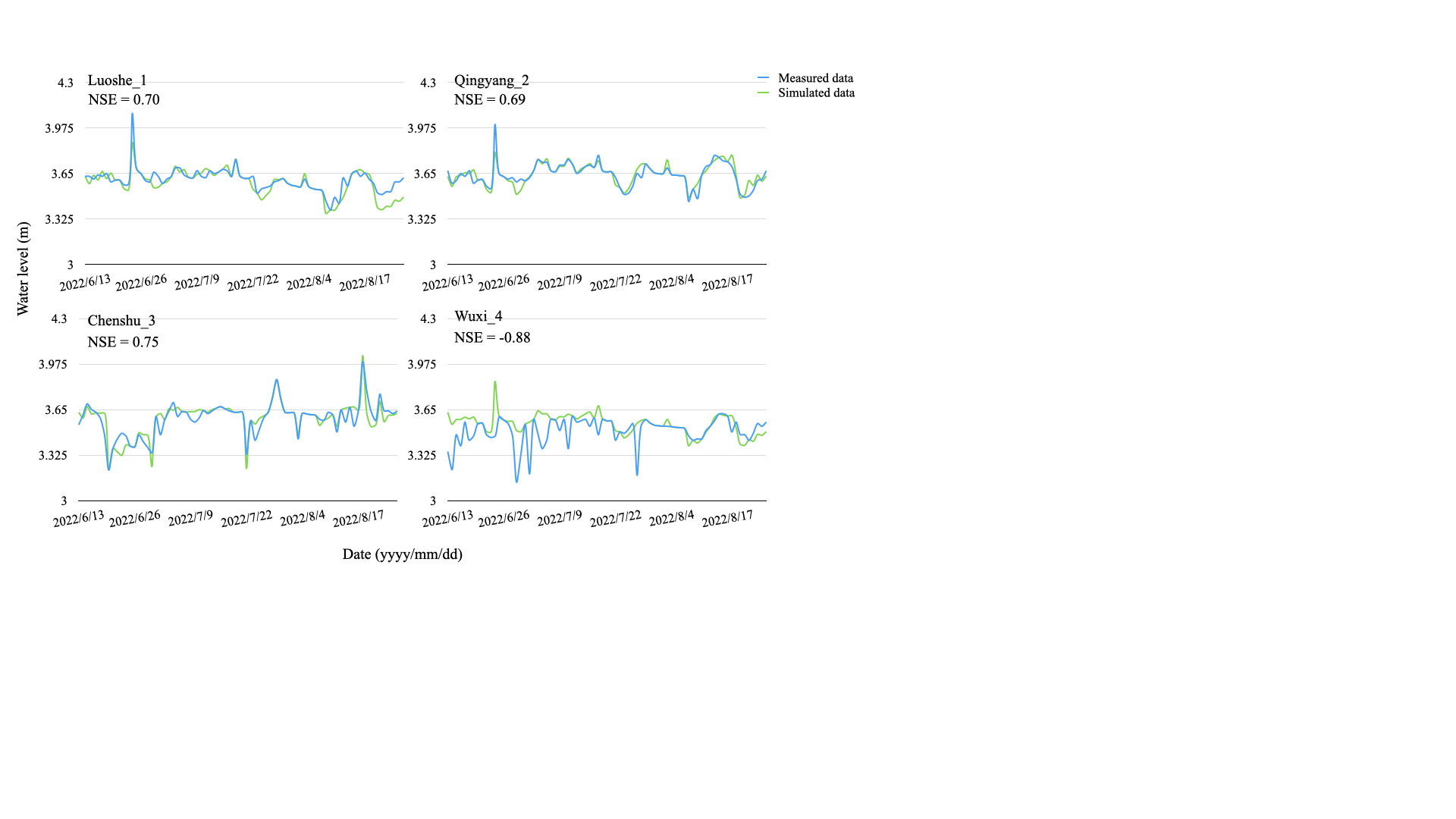


**FIG S6** Measurement and simulation of water level at hydrological stations from June 13 to August 26, 2022 (Validation periods).

FIG S7 Rarefaction curve based on observed species (Sobs) before (Jun. A) and after (Aug. B) the plum rain season.


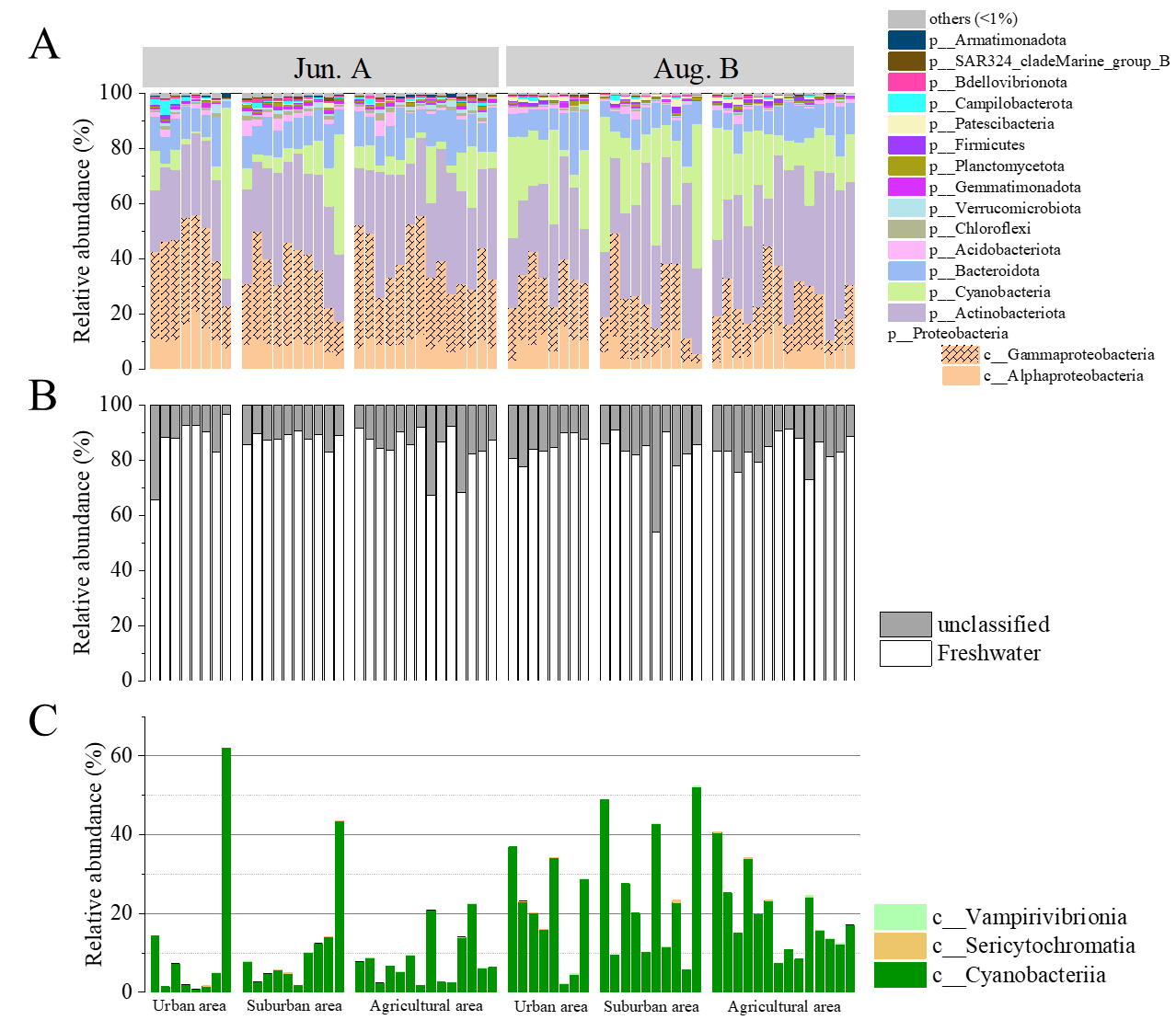


FIG S8 Composition and distributions of bacterioplankton communities before (Jun. A) and after (Aug. B) the Plum rainy season. (A) Composition of bacterioplankton at phylum level, (B) freshwater bacterioplankton, and (C) cyanobacterial communities.

**
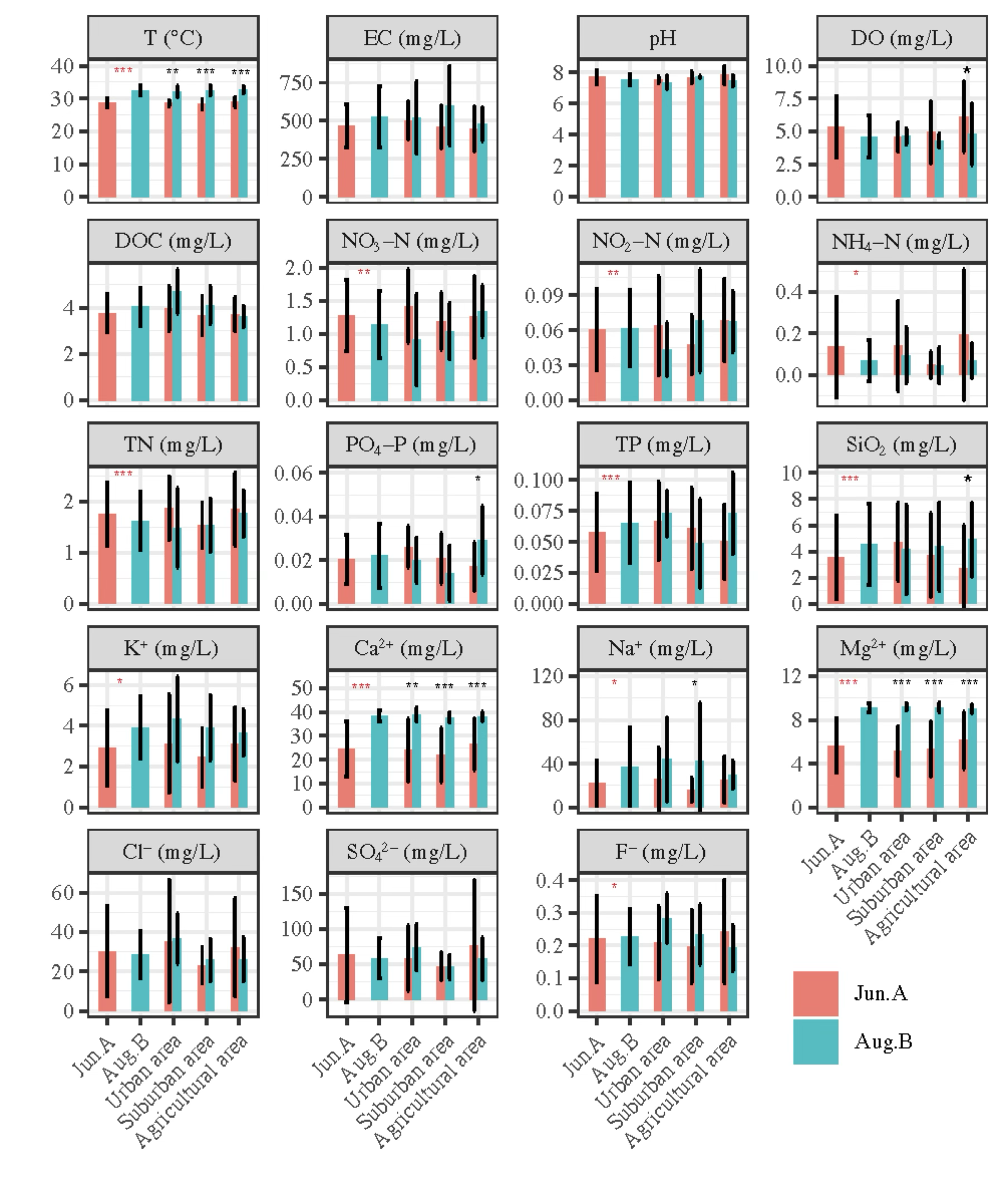
**

FIG S9 Scatterplot showing the content of water chemistry variables before (Jun. A) and after (Aug. B) the Plum Rain season. ****p* < 0.001, ***p* < 0.01, **p* < 0.05.

# Supplementary Tables

Table S1 Information on the top five keystone species in before and after the rainy season.

|  | Topological roles | ASV | Degree | Betweenness centrality | Phylum | Class | Species |
| --- | --- | --- | --- | --- | --- | --- | --- |
| Jun.A | Module hubs | ASV2 | 36 | 1734.31 | Proteobacteria | γ-proteobacteria | g__Candidatus_Methylopumilus |
|  | Connectors | ASV18 | 29 | 2233.65 | Proteobacteria | α-proteobacteria | c__Alphaproteobacteria |
|  | Module hubs | ASV204 | 29 | 1571.99 | Acidobacteriota | Vicinamibacteria | f__Vicinamibacteraceae |
|  | Connectors | ASV4 | 29 | 959.68 | Bacteroidota | Kapabacteria | o__Kapabacteriales |
|  | Module hubs | ASV151 | 28 | 1723.00 | Acidobacteriota | Vicinamibacteria | f__Vicinamibacteraceae |
| Aug.B | Module hubs | ASV566 | 16 | 1749.23 | Verrucomicrobiota | Verrucomicrobiae | Luteolibacter |
|  | Module hubs | ASV26 | 13 | 581.56 | Proteobacteria | γ-proteobacteria | Limnobacter |
|  | Connectors | ASV544 | 10 | 484.03 | Proteobacteria | γ-proteobacteria | Curvibacter |
|  | Module hubs | ASV103 | 8 | 637.00 | Proteobacteria | α- proteobacteria | f__Sphingomonadaceae |
|  | Connectors | ASV453 | 8 | 259.69 | Cyanobacteria | Cyanobacteria | Cyanobium_PCC-6307 |

Note: In the species column, the letter before __ means that the species designation follows the designation under the classification of c__ class, o__ order, f__ family, and g__ genus.

Table S2 Difference of correlation between land use types and water chemistry before and after the Plum rainy season.

|  | Cropland | Forest | Grassland | Water | Imperious surface |
| --- | --- | --- | --- | --- | --- |
| *T* | 0.16/0.06 | -0.15/0.27 | -0.30/-0.17 | -0.14/0.09 | -0.07/-0.05 |
| EC | -0.13/-0.02 | -0.18/**-0.38*** | 0.02/0.20 | 0.24/-0.07 | 0.24/-0.04 |
| pH | 0.09/0.02 | **-0.39***/0.04 | -0.18/0.29 | -0.25/0.06 | -0.06/-0.15 |
| DO | 0.08/-0.16 | -0.16/-0.22 | -0.26/0.08 | -0.26/0.15 | -0.12/0.09 |
| DOC | 0.34/-0.06 | -0.28/-0.1 | **-0.40***/-0.03 | **-0.45***/-0.10 | -0.19/0.09 |
| NO_3_-N | -0.08/-0.11 | -0.22/-0.20 | 0.14/0.12 | 0.23/0.20 | 0.02/0.10 |
| NO_2_-N | -0.06/0.20 | 0.10/-0.34 | -0.04/-0.19 | 0.21/-0.10 | 0.14/-0.09 |
| NH_4_-N | -0.28/0.04 | -0.30/-0.19 | 0.19/0.06 | **-0.51**/-0.46**** | 0.26/-0.02 |
| TN | -0.00/-0.05 | **-0.46****/-0.31 | -0.02/0.06 | -0.08/0.13 | 0.04/0.08 |
| PO_4_-P | 0.09/0.03 | -0.01/-0.09 | 0.12/0.03 | 0.07/0.03 | -0.05/0.02 |
| TP | 0.12/0.00 | **-0.37***/-0.32 | 0.01/-0.00 | -0.09/-0.17 | -0.08/0.10 |
| SiO_2_ | **0.38***/0.27 | **0.42***/-0.29 | -0.29/-0.21 | 0.09/0.07 | -**0.41***/-0.21 |
| K^+^ | 0.19/-0.05 | -0.21/**-0.55**** | -0.01/0.09 | **-0.38***/-0.25 | -0.11/0.09 |
| Ca^2+^ | 0.16/-0.07 | -0.06/**-0.45*** | -0.12/-0.00 | -0.31/0.12 | -0.17/0.14 |
| Na^+^ | 0.16/-0.06 | -0.32/**-0.42*** | -0.03/0.04 | -0.35/-0.18 | -0.09/0.10 |
| Mg^2+^ | 0.21/-0.26 | -0.11/-0.04 | -0.11/0.23 | **-0.36***/-0.27 | -0.19/0.22 |
| SO_4_^2-^ | 0.16/-0.03 | **-0.39*/-0.39*** | -0.21/-0.02 | -0.23/-0.14 | -0.13/0.07 |
| F^-^ | 0.17/0.07 | -0.35/-0.27 | -0.17/-0.09 | **-0.43***/0.03 | -0.13/-0.04 |
| Cl^-^ | 0.16/0.08 | **-0.50**/-0.36*** | -0.16/0.06 | -0.26/0.28 | -0.14/0.10 |

Note: Bolding means significant correlation

1. https://github.com/OpenGene/fastp [↑](#footnote-ref-1)
2. http://www.cbcb.umd.edu/software/flash [↑](#footnote-ref-2)
3. https://www.tba.gov.cn/slbthlyglj/upload/35f9f612-700d-4fb9-b188-6aeb3aee2570.pdf [↑](#footnote-ref-3)
